# A Biocompatible and Self-Healable 3D-Printed Bidirectional Hydrogel Actuator with Needle Injectability

**DOI:** 10.1021/acsami.5c14232

**Published:** 2025-10-02

**Authors:** Kai-Ruei Yang, Qian-Pu Cheng, Shan-hui Hsu

**Affiliations:** Institute of Polymer Science and Engineering, 33561 National Taiwan University, Taipei 106319, Taiwan, Republic of China

**Keywords:** hydrogel actuator, 3D printing, bidirectional actuation, self-healing, *N*-isopropylacrylamide

## Abstract

Multifunctional hydrogels are highly desirable for emerging material applications, particularly for biocompatible hydrogel actuators. However, integrating toughness, self-healing, and reversible bidirectional actuation into a biocompatible actuator remains challenging. Herein, a 3D-printable and biocompatible bilayer hydrogel actuator with reversible bidirectional actuation is developed using a new poly­(*N*-isopropylacrylamide)-gelatin methacryloyl (PNIPAM-GelMA; “PNG”) hydrogel as the active layer. The photo-cross-linked PNG hydrogel shows self-healing ability as well as good elasticity (storage modulus ∼13 kPa) and toughness (linear viscoelastic range up to 240% shear strain). Small-angle X-ray scattering analysis for the microstructure of PNG reveals the presence of dynamic PNIPAM clusters composed of interlocking PNIPAM side chains, accounting for the self-healing behavior of the PNG hydrogel. The 3D-printed bilayer actuator with PNG as the active layer and GelMA as the passive layer exhibits bidirectional actuation and fine needle injectability. Moreover, pairing the PNG active layer with a self-healable passive layer (e.g., polyurethane-GelMA composite hydrogel) gives rise to a self-healable actuator. This actuator, repaired upon cutting, retains significant bidirectional bending angles (∼380° at 37 °C; ∼−270° at 25 °C). The multifunctional PNG system effectively addresses key limitations of current biocompatible hydrogel actuators by integrating toughness, autonomous self-healing ability, and reversible bidirectional actuation, offering substantial progress in developing actuators for biomedical applications.

## Graphical abstract

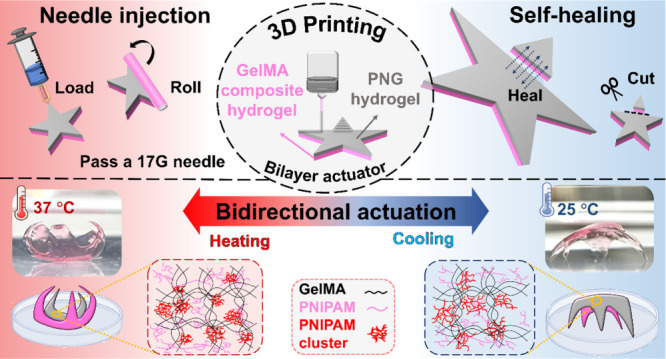

## 1 Introduction

Hydrogels are soft materials comprising a three-dimensional (3D) network structure formed by hydrophilic polymers encapsulating water molecules.^1^ Hydrogels can be created through physical cross-linking or chemical cross-linking.^2^ In recent years, functional hydrogels have gained attention, offering properties like self-healing, stimuli-responsiveness, and 3D printability.^3^ These advanced hydrogels have been explored in diverse applications including soft actuators,^4^ drug delivery system,^5^ and biomimetic tissue constructs.^6^ In particular, stimuli-responsive hydrogels that undergo reversible swelling or contraction in response to environmental cues can be leveraged as actuating materials.^4^ The fabrication method, for instance, layer-by-layer assembly^7^ and extrusion-based 3D printing,^8^ significantly influences the performance and potential applications of hydrogel actuators. Meanwhile, 3D-printed biomimetic hydrogel actuators are commonly used in grippers and soft robot, which often lack biocompatibility.^8^ Developing biocompatible hydrogel actuators for biomedical applications offers realistic simulation platforms for contractile biological tissues including artificial muscles^6^ and artificial blood vessels.^9^ These biocompatible hydrogel actuators with contractile properties contribute to the progress of drug release^10^ and customized tissue engineering.^11^ To fully harness the functional versatility of hydrogel actuators, it is essential to explore how microstructural and anisotropic design strategies affect actuation performance, particularly in achieving reliable, tunable, and complex motions under physiological conditions.

Hydrogel actuators can achieve complicated deformations by incorporating anisotropic structures, such as bilayered, gradient, patterned, or oriented configurations, which have been extensively investigated.^12^ These actuators are often composed of stimuli-responsive polymers,^13^ including pH-responsive poly­(acrylic acid),^14^ humidity-responsive poly­(ethylene glycol) diacrylate,^15^ and thermoresponsive poly­(*N*-isopropylacrylamide) (PNIPAM).^16^ In particular, PNIPAM exhibits a lower critical solution temperature (LCST) of approximately 32 °C, above which the hydrogel network contracts and expels water.^17^ The temperature-induced volume change enables PNIPAM hydrogel to serve as a widely used active layer in bilayer hydrogel actuators. While PNIPAM can be used to create actuators, its direct use in extrusion-based 3D printing for actuator fabrication is challenging due to the lack of injectability and gelling properties of PNIPAM.^13^ Nevertheless, printing of PNIPAM is possible by incorporating other printable polymers as an ink in an extrusion printing system or using the strategy of gel-in-gel printing.^18,19^ Recently, a biocompatible PNIPAM-gelatin methacryloyl (GelMA) composite hydrogel was 3D-printed as actuators.^11^ Despite these advancements, challenges remain in optimizing the composition, improving printability, and enhancing actuation performance to achieve scalable fabrication and robust functionality. Beyond fabrication considerations, it is equally critical to address the mechanical and functional durability of hydrogel actuators for biomedical uses.

Hydrogel actuators face several intrinsic limitations, including brittleness, weak mechanical strength, low durability, and slow response times under cyclic deformation, which hinder their practical deployment in spite of their broad functional applicability.^20^ These shortcomings are especially problematic in biomedical scenarios, where actuators must withstand frequent mechanical loading while maintaining reliable performance.^21^ Efforts to overcome the limitations have focused on developing hydrogels with enhanced elasticity, toughness, and functional durability.^22^ The integration of self-healing capability has emerged as a promising route to enhance the operational lifespan of hydrogel actuators by facilitating autonomous repair of internal or surface damage.^23^ Various self-healing hydrogel systems have been engineered by incorporating dynamic bonds, such as hydrogen bonding, ionic interactions, or reversible covalent linkages, which allow fractured networks to reform spontaneously.^24^ A few PNIPAM-based self-healing systems, including those utilizing hydrogen-bonded network or boronate ester cross-linking, demonstrate good mechanical recovery and preserved actuation performance.^25,26^ Aside from mechanical resilience, another key challenge is achieving programmable, complex actuation behaviors to broaden the functional scope of hydrogel actuators in biomedical contexts. Recent approaches have explored biomimetic hydrogel actuators capable of complex deformation patterns achieved through multilayer architectures, anisotropic reinforcements, or multistimuli-responsive designs.^27,28^ While these methods enable sophisticated motion patterns, they often involve complicated fabrication steps and can compromise system stability, an issue particularly relevant for biomedical devices that demand simplified yet multifunctional material systems.

Currently reported self-healing PNIPAM-based hydrogel actuators are confined to relatively simple linear motion, which limits their functional versatility. Although multifunctional actuators are increasingly demanded in the biomedical field, those combining toughness, self-healing, and reversible bidirectional actuation remain to be scarce, particularly those with thermoresponsive design.^29^ In this study, we address these challenges by developing a tough, 3D-printable PNIPAM-GelMA (PNG) composite hydrogel that uniquely retains its self-healing capability after photo-cross-linking. While a photo-cross-linked PNIPAM homopolymer hydrogel (20 wt % solid content) was found to exhibit intrinsic self-healing behavior, it was unsuitable for actuator fabrication due to high viscosity and low moldability. The moldability was significantly enhanced by incorporating GelMA (4 wt %) into the PNIPAM formulation, leading to a new self-healable and 3D-printable PNIPAM-GelMA (PNG) hydrogel. Moreover, the PNG hydrogel exhibited unique rheological property with a broad linear viscoelastic region (LVR) between 0% and 240% strain, indicating high elasticity and toughness. Small-angle X-ray scattering (SAXS) unveiled a mesoscale spherical structure of PNG, which is associated with PNIPAM cluster formation through side-chain interlocking. The side chains of PNIPAM may reinterpenetrate after damage and interlock to reconstruct clustering features for self-healing. The newly developed PNG hydrogel was 3D-printed as the tough and self-healable active layer of a PNG/GelMA bilayer actuator. The actuator exhibited reversible bidirectional actuation behavior in water between 37 and 25 °C. Notably, the actuator demonstrated the additional feature of needle injectability. When combining with a self-healable passive layer, the PNG bilayer actuator further provided the integrated self-healing capability. The versatile PNG hydrogel platform developed in the work overcomes some key limitations of prior hydrogel actuators by integrating high toughness, self-healing, and bidirectional motion, offering significant potential for advanced biomedical actuator applications.

## 2 Materials and Methods

### 2.1 Synthesis of Gelatin Methacryloyl (GelMA)

Carbonate-bicarbonate (CB, 0.25 M) buffer was prepared by dissolving 0.795 g sodium carbonate (Riedel-de Haën, Germany) and 1.465 g sodium bicarbonate (Sigma-Aldrich, USA) in 100 mL of deionized water (DI water). GelMA was prepared by reacting gelatin (type A, 300 Bloom, Sigma-Aldrich, USA) with methyl acrylic anhydride (MAA, Sigma-Aldrich) following a standard protocol.^30^ Briefly, gelatin (10 w/v%) was dissolved in 0.25 M CB buffer (100 mL) at 45 °C under constant stirring. MAA (0.2 mL per gram of gelatin) was added dropwise to the gelatin solution and allowed to react for 90 min with continuous stirring. GelMA with a DS of approximately 95% and 47% was obtained by adjusting the feed ratio to 0.2 and 0.05 mL/g (MAA/gelatin), respectively. After the reaction, the mixture was transferred into a dialysis membrane (molecular weight cutoff = 12–14 kDa, Viskase Co., USA) and dialyzed against DI water at room temperature for 72 h to remove unreacted reagents. The dialyzed solution was then lyophilized to obtain the GelMA product. The degree of substitution (DS) of the resulting GelMA was determined by proton nuclear magnetic resonance (1H NMR) spectroscopy using a Bruker Avance III-500 MHz instrument in deuterium oxide (D_2_O, Sigma-Aldrich). The aromatic proton signal at δ ∼ 7.2 ppm (from phenylalanine, tyrosine, and tryptophan residues) was used as an internal reference. Spectra were normalized to this peak, and the decrease of the amine-related resonance at δ ∼ 3.01 ppm was integrated to calculate DS.

### 2.2 Preparation and Characterization of PNIPAM-GelMA (PNG) Hydrogels

PNG hydrogels were fabricated via free-radical polymerization under UV irradiation. The total solid content in the mixture of *N*-isopropylacrylamide (NIPAM, 99%, Thermo Fisher Scientific, USA) monomer (19–15 wt %) and GelMA (1–5 wt %) solutions was fixed at 20 wt % and was mingled with the photoinitiator 2,2-azobis­(2-methyl-N-(2-hydroxyethyl) propionamide) (VA-086, Wako Chemicals GmbH, Germany) for 2 wt % with respect to total solids. The resulting precursor solution was dispensed into a transparent polystyrene mold at 4 °C for 1 h and irradiated with UV light (360–480 nm, 22.4 mW/cm^2^ at a 5 cm distance) for 10 min to form cross-linked network. Fourier transform infrared (FT-IR) spectroscopy was employed to confirm the formation of PNIPAM-GelMA networks in both the un-cross-linked precursor and the cured hydrogel. Dried samples were finely ground with potassium bromide (KBr) and pressed into pellets. FT-IR (PerkinElmer Spectrum, USA) spectra were recorded with a resolution of 4 cm^–1^ over the wavenumber range of 400–4000 cm^–1^.

The self-healing ability of PNG hydrogel was observed by placing the PNG precursor solution into a transparent polystyrene mold and irradiation with UV light for 10 min to form PNG hydrogel. Subsequently, PNG hydrogel was chopped into small fragments within millimeters and loaded into a round-shaped mold. PNG hydrogel fragments adapted into the round shape at 25 °C within 12 h.

The gel fractions of the PNG hydrogel were determined to assess the degree of chemical cross-linking. For this, hydrogel samples were first dried (24 h lyophilization) and weighed to obtain the initial dry weight (W_i_). Each sample was then immersed in DI water at 25 °C for 24 h to extract any unreacted components, after which it was dried again (another 24 h by lyophilization) to determine the final dry weight (W_f_). The gel fraction was calculated by the following equation:

$$\mathrm{Gel}\;\mathrm{fraction}\left(\%\right)=\left(W_{f}/W_{i}\right)\times 100$$ 1

The swelling behavior of the PNG hydrogels was evaluated by gravimetric analysis at 25 °C, over time and quantified as equilibrium swelling ratios. The preweighed hydrogel samples (W_p_) were submerged for a specified duration. At predetermined time intervals, samples were removed, gently blotted to eliminate surface water, and weighed to determine the swollen weight (W_t_). The swelling ratio at time t was calculated using the formula:

$$\mathrm{Swelling}\;\mathrm{ratio}\left(\%\right)=\left(W_{t}-W_{p}\right)/W_{p}\times 100$$ 2

The remaining ratio was related to the thermoresponsive deswelling behavior after immersion in 37 °C DI water. The swollen weight (W_s_) was recorded after eliminate surface moisture, and W_d_ denoted the equilibrium weight for the deswelled hydrogel at a given time.

$$\mathrm{Remaining}\;\mathrm{ratio}\left(\%\right)=\left(W_{d}/W_{s}\right)\times 100$$ 3

### 2.3 Rheological Characterization of PNG Precursor Solutions and Hydrogels

Rheological measurements were carried out using a rotational rheometer (TA Instruments, HR-2) to evaluate the viscoelastic behavior of the PNG system in both the sol (un-cross-linked) and gel (cross-linked) states. All measurements were conducted at 25 °C unless otherwise specified. A 20 mm parallel-plate geometry was used for most tests, while a 40 mm, 2° cone–plate configuration was employed for the temperature-dependent and viscosity analyses. All tests were conducted to acquire storage (*G*′) and loss (*G*″) moduli. For temperature-dependent testing, an oscillatory temperature sweep (1 Hz, 1% strain) was performed while heating the sample from 5 to 40 °C at 1 °C/min. Steady shear rheology was conducted to measure the viscosity as a function of shear rate, by ramping the shear rate from 0.3 s^–1^ to 100 s^–1^. The time sweep was researched at 1% strain and 1 Hz during *in situ* photopolymerization (22.4 mW/cm^2^). The frequency sweep was conducted at 25 °C and 1% strain over a frequency range of 0.1–50 Hz. The dynamic strain sweep was carried out on the cured hydrogels at 1 Hz by increasing the oscillatory strain from 0.1% up to 1200%. The self-healing capacity was evaluated based on results from a strain sweep test. Damage-healing cycles were performed by applying oscillatory strain steps at a frequency of 1 Hz, alternating between 1% and a strain value of n% (where n% corresponds to the strain at which the gel-to-sol transition occurs). Self-healing efficiency was defined as the percent recovery of *G*′ after three damage-healing cycles.

### 2.4 SAXS Analysis of PNG Hydrogel

Temperature-dependent SAXS measurements were carried out on a representative PNG hydrogel formulation and its un-cross-linked precursor solution, as well as on pure GelMA and NIPAM hydrogels for comparison. All measurements were performed at beamline 13A of the Taiwan Light Source (National Synchrotron Radiation Research Center, Hsinchu, Taiwan). The X-ray energy was 8 keV, providing a scattering vector q-range of approximately 0.014–1.4 nm^–1^. Scattering profiles were collected at temperatures from 10 to 40 °C, with each sample held at the target temperature for 10 min before measurement to ensure thermal equilibrium.

The SAXS curves were modeled using the SasView5.0.6 software. The following model function was used to fit the scattering curves from SAXS experiments:

$$I\left(q\right)=I_{\mathrm{Sp}}\left(q\right)S_{\mathrm{HS}}\left(q\right)+I_{\mathrm{OZ}}\left(q\right)+I_{\mathrm{bkg}}$$ 4

where I­(q) indicate the overall intensity after data reduction from the experiment. I_bkg_ denotes the background.

I_Sp_(q) is the sphere model with a uniform scattering length density, defined as

$$I_{\mathrm{Sp}}\left(q\right)=\frac{\mathrm{sacle}}{V}\cdot {[3V_{\mathrm{Sp}}\left(\Delta \rho \right)\cdot \frac{\sin\left(\mathrm{qr}\right)-\mathrm{qr}\cos\left(\mathrm{qr}\right))}{(\mathrm{qr}{)}^{3}}]}^{2}$$ 5

$$\Delta \rho =\rho_{\mathrm{Sp}}-\rho_{\mathrm{solvent}}$$ 6

where r is the radius of the cluster. V_Sp_ calculated from V = 4πr^3^/3 represents the volume of the clusters. ρ_Sp_ and ρ_solvent_ are the scattering length density (SLD) of the cluster and the solvent, respectively. According to the literature, SLD of water is ρ_Water_ = 9.46 × 10^–6^ Å^–2^.

S_HS_(q) is the hard-sphere structure factor which applies the Percus–Yevick approximation to model the correlation between the particles:

$$S_{\mathrm{HS}}\left(q\right)=\frac{1}{1+24\eta G\left(2R_{\mathrm{HS}}q\right)/2R_{\mathrm{HSq}}}$$ 7

where R_HS_ is the hard-sphere radius which denotes as a half of the center to center distance between the particles. η is the hard-sphere volume fraction that shows the correlating fraction of particles, and G is defined below:

$$G\left(x\right)=\gamma \frac{\mathrm{sinx}-\mathrm{xcosx}}{x^{2}}+\kappa \frac{2\mathrm{xsinx}+\left(2-x^{2}\right)\mathrm{cosx}-2}{x^{3}}+\varepsilon \left[\frac{-x^{4}\mathrm{cosx}+4\left(3x^{2}-6\mathrm{cosx}+\left(x^{3}-6\mathrm{xsin}x+6\right)\right)}{x^{5}}\right]$$ 8

where $\gamma =\frac{{\left(1+2^{\eta }\right)}^{2}}{{\left(1+\eta \right)}^{4}}\kappa =\frac{‐6\eta {\left(1+\eta /2\right)}^{2}}{{\left(1-\eta \right)}^{4}},\;\mathrm{and}\;\varepsilon =\frac{\gamma \eta }{2}$

I_OZ_(q) denotes the Ornstein–Zernike function, describing the average local chain distance between GelMA and PNIPAM chains as well as the average distance between PNIPAM clusters

$$I_{\mathrm{OZ}}(q)=\frac{I_{\mathrm{scale}}^{\prime }}{{\left(1+\Xi^{2}q^{2}\right)}^{2}}+\frac{I_{\mathrm{scale}}^{\prime \prime }}{1+\xi^{2}q^{2}}$$ 9

where I_scale_^′^ and I_scale_^″^ are the scaling factors. Ξ is the correlation length of the local “frozen-in” clusters introduced by the strong physical associations and ξ is the correlation length of thermal fluctuations. These fluctuations give rise to the scattering at the high-q region.

### 2.5 Needle Injectability of UV-Cured 3D-Printed PNG Hydrogel

The PNG′4 precursor solution was poured into a cylindrical syringe and kept for 12 h at 4 °C. A commercial 3D bioprinter (Regenovo, Bio-Printer-WS, China) was used to extrude the gel-like ink directly through a needle-shape nozzle (either 80 or 210 m in diameter). The nozzle was maintained at 4 °C during printing, with an extrusion pressure of 480 kPa and a printing speed of 2 mm/s. A sheet-like construct consisting of two stacked layers was printed on a platform held at 4 °C, and it was subsequently irradiated with UV light for 10 min to ensure network cross-linking. After UV curing, the printed hydrogel sheet was rolled into a cylinder and incubated in air at 37 °C for 5 min. The resulting cylindrical hydrogel was then placed into a 5 mL-syringe half-filled with water and extruded through the syringe needle (17G). The initial total volumes of the 3D printed sheet (V_i_) were determined by measuring its dimensions, with the height measured after photo-cross-linking. The total volumes of the constructs after placing in 37 °C air (V_fh_) and injecting through the 17G needle were also calculated and divided by V_i_ to obtain the volume ratios.

### 2.6 Actuation Behavior of PNG/GelMA Bilayer Hydrogels

The PNG**/**GelMA bilayer hydrogel actuators were fabricated using a sequential casting method to construct distinct passive and active layers. Initially, 0.3 mL of GelMA DS 47% solutions (5, 7.5, or 10 wt %) each containing VA-086 at 2 wt % of the total solid content, were cast into a strip-shaped mold and irradiated with UV light for 5 min to form the passive layer. Subsequently, 0.3 mL of a mixture composed of NIPAM (16 wt %), GelMA DS 47% (4 wt %), and VA-086 (2 wt % of the total solid content) was cast atop the cured passive layer and cross-linked under UV irradiation for 10 min to generate the active PNG′4 layer. As a control, a bilayer hydrogel actuator (GN2-GelMA) based on a previously reported formulation^11^ was prepared, comprising an active layer of NIPAM (7.5 wt %), GelMA DS 95% (2.5 wt %), and VA-086 (2 wt % of the total solid content) and a passive layer of GelMA (7.5 wt %) with the same photoinitiator content. All bilayer hydrogel strips were fabricated to dimensions of 40 mm × 6 mm × 2 mm, with the active to passive layer thickness ratio maintained at about 1:1. The bending direction was defined by the relative positions of the active and passive layers: bending toward the GelMA layer (passive layer) was designated as a negative bending angle, whereas bending toward the PNG layer (active layer) was designated as a positive bending angle. Bending angles were quantified by measuring the angular displacement between the initial and terminal vector along the longitudinal edge of the hydrogel. The thermally induced actuation behavior was characterized in DI water baths maintained at 25 and 37 °C. To optimize actuation performance, three different GelMA concentrations in the passive layer were compared. Furthermore, cyclic bending tests involving five sequential thermal transitions were conducted in both DI water and phosphate-buffered saline (PBS, Sigma-Aldrich) to assess the reversibility and mechanical robustness of the bilayer hydrogels. For long-term stability, additional fatigue tests were carried out in PBS for 50 consecutive thermal cycles. Stepwise heating and cooling experiments were also performed at 25, 28, 31, 34, 37, and 40 °C DI water, during which bending angles and response times were recorded at each equilibrium temperature. All optical images were captured with the hydrogels fully submerged in water, except for images recorded immediately after UV curing. To enhance visual differentiation between layers, the passive GelMA layer was stained pink using Safranin-O (Sigma-Aldrich, USA) dye prior to assembly.

The fabrication of the star-like and leaf-like hydrogel actuators involved sequential 3D printing of stacked layers (two layers for star-like actuators; 12 layers for leaf-like actuators). First, GelMA ink (matching the passive layer composition) was printed at 24 °C through a 210 μm needle-like nozzle at a pressure of 450 kPa and a printing speed of 2 mm/s. Following this, PNG′4 ink, preconditioned by refrigeration at 4 °C for 6 h to attain a gel-like consistency, was printed directly onto the GelMA layer at 4 °C under 300 kPa pressure and a speed of 2.5 mm/s. The star-like actuators of two different sizes consisted of two stacked layers. The bigger star had a lateral size approximately ∼22 mm, and the smaller star had a lateral size of ∼9 mm. Both stars were ∼0.5 mm thick. The leaf-like construct with fine edges was printed with six sequential layers of GelMA followed by six layers of PNG′4 under the same parameters. The CAD model of the leaf-like actuator was designed with dimensions of 25 mm × 14 mm × 2.52 mm, and the actual printed hydrogels closely matched the design, with measured dimensions of approximately 24.7 mm in length, 13.8 mm in width, and 2.49 mm in thickness. After printing, the constructs were irradiated with UV light for 10 min to complete cross-linking. Additionally, strip-shaped bilayer hydrogels were fabricated by sequentially printing four GelMA layers followed by four PNG′4 layers along either the X- or *Y*-axis. The CAD model was designed with dimensions of 40 mm × 6 mm × 2 mm. The actual printed hydrogels closely matched the design, with measured dimensions of approximately 37.4 mm in length, 5.8 mm in width, and 1.8 mm in thickness. Top-view optical microscopy was performed on UV-cross-linked X- and *Y*-axis prints to examine layer alignment and surface porosity. An additional strip-shaped bilayer hydrogel was printed using an 80 μm nozzle; the GelMA and PNG′4 layers were deposited as nine layers each to obtain a size comparable to the 210 μm nozzle prints. GelMA ink was printed at 24 °C and 500 kPa in 2 mm/s, while PNG′4 ink was printed at 4 °C and 340 kPa in 2 mm/s. The actuation behaviors of all printed constructs were assessed by immersing them alternately in 37 and 25 °C water baths. Photographs were taken to record the deformation and recovery processes during temperature transitions.

### 2.7 Cell Proliferation in Hydrogels and Degradation of Hydrogels

The study utilized the C2C12 myoblast cell line, originating from mouse musculus, for conducting three-dimensional cell culture experiments. The cells were maintained in Dulbecco’s Modified Eagle Medium (DMEM, Gibco, USA), enriched with 10% fetal bovine serum (FBS, Gibco, USA) and 1% penicillin-streptomycin (Caisson, USA), under controlled conditions of 37 °C and 5% CO_2_ in a humidified incubator. To evaluate the cell viability within the PNG and GelMA hydrogel matrices, live/dead staining assays were carried out. After 3 h of incubation postfabrication, the cell-embedded hydrogels were washed thoroughly with PBS solution to remove excess medium. The hydrogels were then stained with a mixture of calcein AM and ethidium homodimer-1 (LIVE/DEAD Viability Kit, Invitrogen, USA) for approximately 15 min. Fluorescence imaging was then conducted using a Leica DM IRB microscope, employing excitation wavelengths of 488 and 514 nm for visualization of live and dead cells, respectively. The proliferation of encapsulated C2C12 cells was assessed at various time points (0, 1, 3, 7, and 14 days) using the Cell Counting Kit-8 (CCK-8, Sigma-Aldrich, Japan). For cell embedding, C2C12 cells (6 × 10^6^ cells/mL) were mixed with the respective PNG and GelMA precursor solutions. The mixtures were then transferred to a 24-well plate and subjected to UV light exposure for 10 min to form cross-linked hydrogels. Afterward, the hydrogels were rinsed three times with PBS and cultured in DMEM at 37 °C. At each time point, the hydrogels were incubated in a 1:10 dilution of CCK-8 solution for 3 h, and the absorbance at 450 nm was measured using a microplate reader (SpectraMax M5, Molecular Devices, USA). The proliferation rate of cells was determined based on the absorbance values, reflecting the metabolic activity of the cells within the hydrogels.

Hydrogel degradation *in vitro* was evaluated under the physiological condition. PNG hydrogels and control GelMA hydrogels (7.5 wt % GelMA) were incubated in PBS at 37 °C for up to 28 days. Prior to immersion, samples were lyophilized for 24 h to obtain their initial dry weight (W_di_). At predetermined time points (after 7, 14, 21, and 28 days of incubation), samples were removed from PBS, rinsed with DI water three times, lyophilized, and weighed (W_df_). The remaining weight of hydrogels was calculated by the following equation:

$$\mathrm{Remaining}\;\mathrm{weight}\left(\%\right)=W_{\mathrm{df}}/W_{\mathrm{di}}\times 100$$ 10

### 2.8 Needle Injectability and Bidirectional Actuation of the 3D-Printed Hydrogel Actuator

The aforementioned smaller star-like bilayer hydrogel actuator made by 3D printing was used for the needle injectability study. The star-like hydrogel was rolled into a cylinder and placed in 37 °C air for 5 min. Subsequently, the cylindrical hydrogel was loaded into a 5 mL-syringe half-filled with water and extruded through the syringe-needle (17G) into a water bath at 37 °C. The star-like hydrogel was transferred into a separate DI water bath at 25 °C to examine its shape recovery and actuation behavior. The initial gross volume (V_i_) of the 3D-printed star-like hydrogel was estimated based on dimensional measurements, including the height after UV cross-linking. After placement in 37 °C air, the final gross volume (V_fh_) of the constructs was measured. The volume ratios were calculated by dividing V_fh_ by V_i_ to quantify the extent of volume change during thermal exposure.

### 2.9 Preparation of Self-Healable Passive PUGG Hydrogel

The complete preparation method was depicted in prior literature.^31^ In brief, biodegradable waterborne polyurethane dispersion was synthesized from isophorone diisocyanate (IPDI, Evonik Degussa GmbH, Germany) and two oligomeric diols, poly­(D, l-lactide) (PDLLA) diol (Mn ∼1500 Da) and polycaprolactone (PCL) diol (Mn ∼2000 Da, Sigma-Aldrich, USA) with 2,2-bis­(hydroxymethyl)­propionic acid (DMPA, Sigma-Aldrich, USA) as the self-emulsified chain extender and ethylenediamine (EDA, Tedia, USA) as the final chain extender in water dispersion. The molar ratio of IPDI to oligodiol, DMPA, and EDA was set to 3.52:1:1:1.52.^32^ The PUGG hydrogel was prepared by mixing the polyurethane dispersion with GelMA DS 95% and gelatin. Under conditions of 37 °C, the components were fully dissolved in low-glucose DMEM containing 1% sodium bicarbonate. Subsequently, VA-086 was incorporated into the precursor solution, achieving a final concentration of 1.5 w/v% of the total solid content. In the precursor solution, polyurethane, GelMA DS 95%, gelatin, and VA-086 accounted for 12, 4, 3, and 0.285 wt %, respectively.

### 2.10 Self-Healability and the Bidirectional Actuating Capacity of the Self-Healed 3D-Printed Hydrogel Actuator

To construct a self-healable actuator, a self-healable passive PUGG hydrogel was first prepared. The complete preparation method for the PUGG hydrogel was depicted in prior. The PNG/PUGG bilayer hydrogel actuator was fabricated using a sequential casting method. First, 0.3 mL of the PNG′4 precursor solution (prepared as described previously) was cast into a strip-shaped mold and irradiated with UV light for 10 min to form the active PNG′4 layer. Next, 0.3 mL of the PUGG solution was poured on top of the cured active layer and UV-cured for 1 min to create the passive PUGG layer. The resulting PNG/PUGG bilayer construct was then cut into two pieces. The cut surfaces were carefully realigned and left in contact at either 25 or 37 °C for 20 min to allow self-healing. The actuation behavior of the bilayer hydrogel was evaluated in DI water at 25 and 37 °C. The bending direction and bending angle were defined as described earlier in the text. To characterize the actuation performance, both the intact and self-healed actuators were subjected to five consecutive bending/unbending cycles by alternately transferring them between the 37 and 25 °C water baths. All optical images were captured with the hydrogel fully submerged in water, except for images taken immediately after UV curing and after self-healing. For clear visual distinction between layers, the passive PUGG layer was prestained pink with Safranin-O dye before the bilayer assembly.

A star-like hydrogel actuator was fabricated by a continuous 3D printing process consisting of 12 sequential layers. First, the PUGG ink was printed at 25 °C through a 210 μm nozzle under a pressure of 280 kPa, at a printing speed of 3 mm/s, to form the passive layer consisted of 6 stacked layers. Next, the PNG′4 ink, which had been refrigerated at 4 °C for 6 h to obtain a gel-like consistency, was directly printed at 4 °C onto the underlying layer using a 210 μm nozzle at 300 kPa and 2.5 mm/s to form the active layer composed of 6 stacked layers. The printed star-like construct had a lateral size of ∼22 mm and a thickness of ∼2.5 mm. After printing, the entire structure was exposed to UV light for 10 min to ensure full cross-linking of all layers. To prepare a self-healed sample, one corner of the cured star-like hydrogel actuator was cut off and immediately placed back into contact with the main body. The severed corner was allowed to self-heal for 20 min at room temperature, reattaching to restore the integrity of the star actuator. The actuation behavior of both the intact and self-healed star actuators was then assessed by alternately immersing them in water baths at 37 and 25 °C, inducing cyclic deformation. Photographs were taken throughout these thermal cycles to record the shape deformation and recovery of the star-shaped actuators during heating and cooling transitions.

### 2.11 Statistical Analysis

All experiments were performed in triplicate, and data are presented as the mean ± SD. Statistical analyses were carried out using Student’s *t* test, with *p* < 0.05 considered statistically significant.

## 3 Results

### 3.1 Synthesis and Characterization of GelMA and PNG Hydrogels

Figure S1A illustrates that the synthesis of GelMA was successfully verified through NMR spectroscopy. GelMA with different degrees of substitution (DS) was derived from gelatin by grafting methacrylate groups to varying extents. The characteristic peak at 3.01 ppm, associated with the amine protons (NH_2_) of gelatin, exhibited a marked reduction in GelMA. Based on the diminished peak intensity, approximately 95% and 47% of the amine groups were substituted. Meanwhile, the characteristic peaks appearing at 5.68 and 5.44 ppm were correspond to the vinyl protons introduced by methacryloyl modification.

The structure of PNIPAM-GelMA (PNG) hydrogels is shown in Figure 1. PNG hydrogels were generated after mixing GelMA (DS 95% or 47%) and NIPAM in different proportions followed by photo-cross-linking. Figure S1B presents FT-IR profiles of PNG precursors and hydrogels, highlighting chemical changes after cross-linking. The characteristic peak of C = C stretching at 1600 and 1650 cm^–1^ was evident in the precursor spectrum. After photo-cross-linking, this peak disappeared, confirming the consumption of vinyl groups through radical-mediated addition reactions. Concurrently, the formation of methylene groups at the newly formed cross-linking sites led to increases in peak intensities at 2870–3000 cm^–1^ (C–H stretching) and 1462 cm^–1^ (C–H bending). Meanwhile, the bands at 3400–3200 cm^–1^ (N–H stretching), 1544 cm^–1^ (N–H bending), and 1658 cm^–1^ (C=O stretching) were attributed to PNIPAM. Symmetrical stretching vibrations of methyl groups (−CH­(CH_3_)_2_) in PNIPAM were identified at 1387 cm^–1^ and 1377 cm^–1^. In the PNG (DS 95%) system, carbonyl and amide groups in PNIPAM chains may form hydrogen bonds. In the PNG (DS 47%) system, the unsubstituted hydroxyl and amine groups in GelMA may form additional hydrogen bonds with PNIPAM.

**Figure 1:**
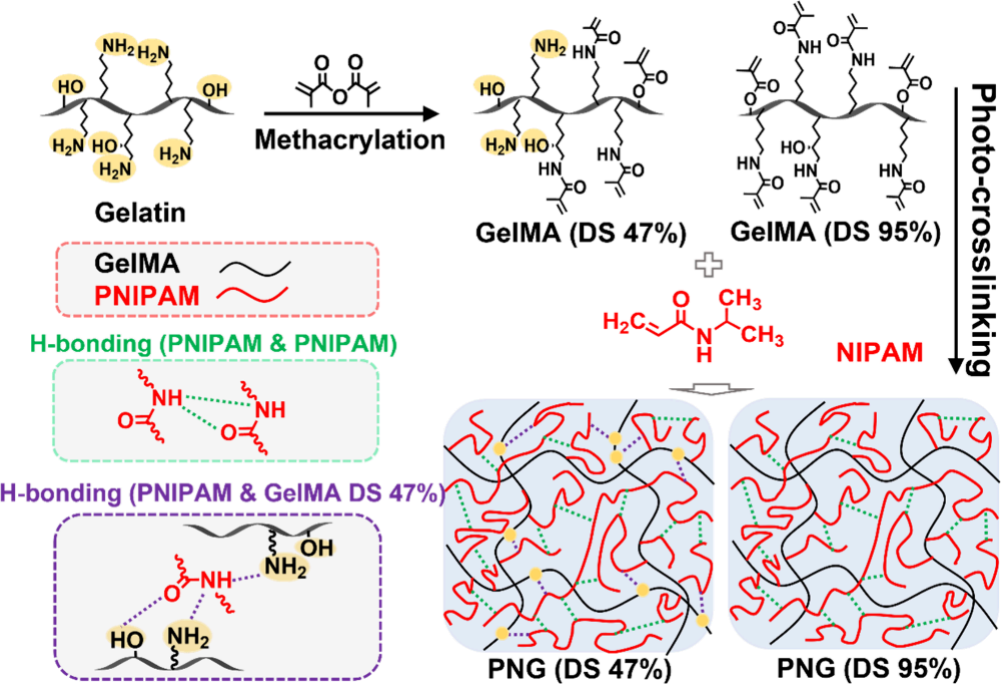
Hypothetic structure diagram of PNIPAM-GelMA (PNG) hydrogels from various ratios of NIPAM and GelMA after photo-cross-linking. Hydrogen bonds between different functional groups are represented by dashed lines.

### 3.2 Optimization of PNG Hydrogels

Fabrication of PNG hydrogels is outlined in Figure 2A. First, a precursor solution was prepared by mixing NIPAM and GelMA at a total solid content of 20 wt %. The precursor solution is then stored at 4 °C to form a reversible physical gel and obtain the injectability. Finally, the physical gel underwent UV irradiation (365 nm) at 4 °C, leading to the formation of the photo-cross-linked PNG hydrogel. The formulas of PNG hydrogel were initially screened by evaluating the flow property of the precursor at 4 °C (i.e., being an injectable physical gel through a 23 G needle, Figure 2B), as well as the self-healing ability (i.e., healing of cut pieces, Figure 2C) of the post-UV PNG hydrogel at 25 °C and the thermoresponsive swelling/deswelling property of the cross-linked structures upon cycles of alternating 25 and 37 °C (Figure 2D).

**Figure 2:**
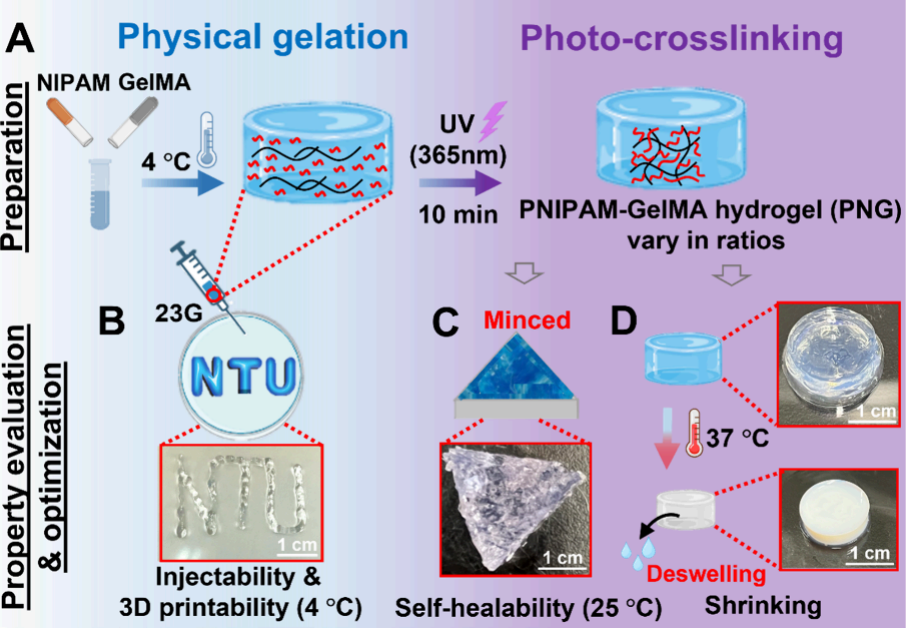
Fabrication of PNG hydrogels and optimization of the formula. (A) PNG hydrogels were prepared from photo-cross-linking the NIPAM-GelMA mixture (a physical gel at 4 °C) under 4 °C. (B) For the mixture, 23G needle injectability was assessed at 4 °C. (C, D) For photo-cross-linked hydrogels (PNG), self-healing ability at 25 °C and thermoresponsive swelling/deswelling properties upon cycles of 25 and 37 °C were assessed.

Table S1 lists the results from formula evaluation. For formulas with DS 95% GelMA, those with less than 3 wt % GelMA failed to form physical gels at low temperatures and those with higher concentrations of GelMA became difficult to inject (e.g., 10 wt % GelMA). The photo-cross-linked hydrogels exhibited self-healing ability, but diminished when GelMA (DS 95%) exceeded 3 wt %. To incorporate GelMA while preserving the self-healing ability, GelMA with a lower DS appeared to be favorable. For formulas with DS 47% GelMA, those containing 5 wt % GelMA displayed adequate self-healability. Following preliminary screening, the groups of PNG3, PNG′3, PNG′4, and PNG′5 were selected and subjected for further optimization, where the swelling ratio and gel fraction were measured with results detailed in Table 1. All four selected PNG hydrogels showed similar thermoresponsive deswelling behavior after immersion in 37 °C water (Figure S2A) as well as robust gel shrinkage-expansion behavior during the deswelling-reswelling cycles (Figure S2B, PNG′4 serving as an example).

**Table 1:** Gel Fraction and Swelling Ratio of Selected PNG Hydrogels

	composition (wt %)				
group	NIPAM	GelMA (DS 95%)	GelMA (DS 47%)	gel fraction (%)	swelling ratio (%)
PNG3	17	3	0	86.93 ± 0.79	116.24 ± 3.18
PNG′3	17	0	3	82.35 ± 3.36	214.82 ± 3.49
PNG′4	16	0	4	79.10 ± 1.39	194.12 ± 3.54
PNG′5	15	0	5	77.13 ± 0.96	177.39 ± 3.67

### 3.3 Rheological Characterization of PNG Precursors and Hydrogels

Table S2 summarizes the rheological characteristics of selected PNG precursors and hydrogels. During the temperature sweep experiment, PNG3, PNG′3, PNG′4, and PNG′5 precursors each can form physical gels at the sol–gel transition temperature, respectively. As shown in Figure 3A, PNG′4 precursor became gel-like when the temperature was below 19.4 °C. To assess printability within the sol–gel transition temperature, steady shear measurements were applied to the PNG precursors. All four PNG precursors exhibited shear-thinning properties, evidenced by the decrease in steady shear viscosity with increasing shear rate, as demonstrated by the case of PNG′4 in Figure 3B. After UV curing for 10 min, PNG hydrogels exhibited a notable rise in storage modulus (*G*′), reaching ∼13 kPa, surpassing the corresponding loss modulus (*G*″), as illustrated in Figure 3C. The frequency sweep experiment confirmed the stability of the PNG hydrogel. For instance, PNG′4 hydrogel exhibited a stable gelation modulus across a frequency range from 0.1 to 50 Hz, as shown in Figure 3D. The strain sweep experiment showed that *G*′ remained closed to *G*″ in all hydrogels when the strain exceeded the damage threshold, particularly in formulas with GelMA DS 47% (PNG′3, PNG′4, and PNG′5), as depicted in Figure 3E and Figure S3B,C. Of particular interest, all four PNG hydrogels exhibited an obvious linear viscoelastic region (LVR). For example, PNG′4 showed a linear viscoelastic region (LVR) between 0% to 240% strain in Figure 3E [the range marked by (i)]. Within LVR, the internal structure of the hydrogel undergoes a reversible deformation under applied strain. The hydrogel is highly elastic and can restore its original configuration without damage once the imposed strain is removed. The broad strain range also indicates toughness for the hydrogel.

**Figure 3:**
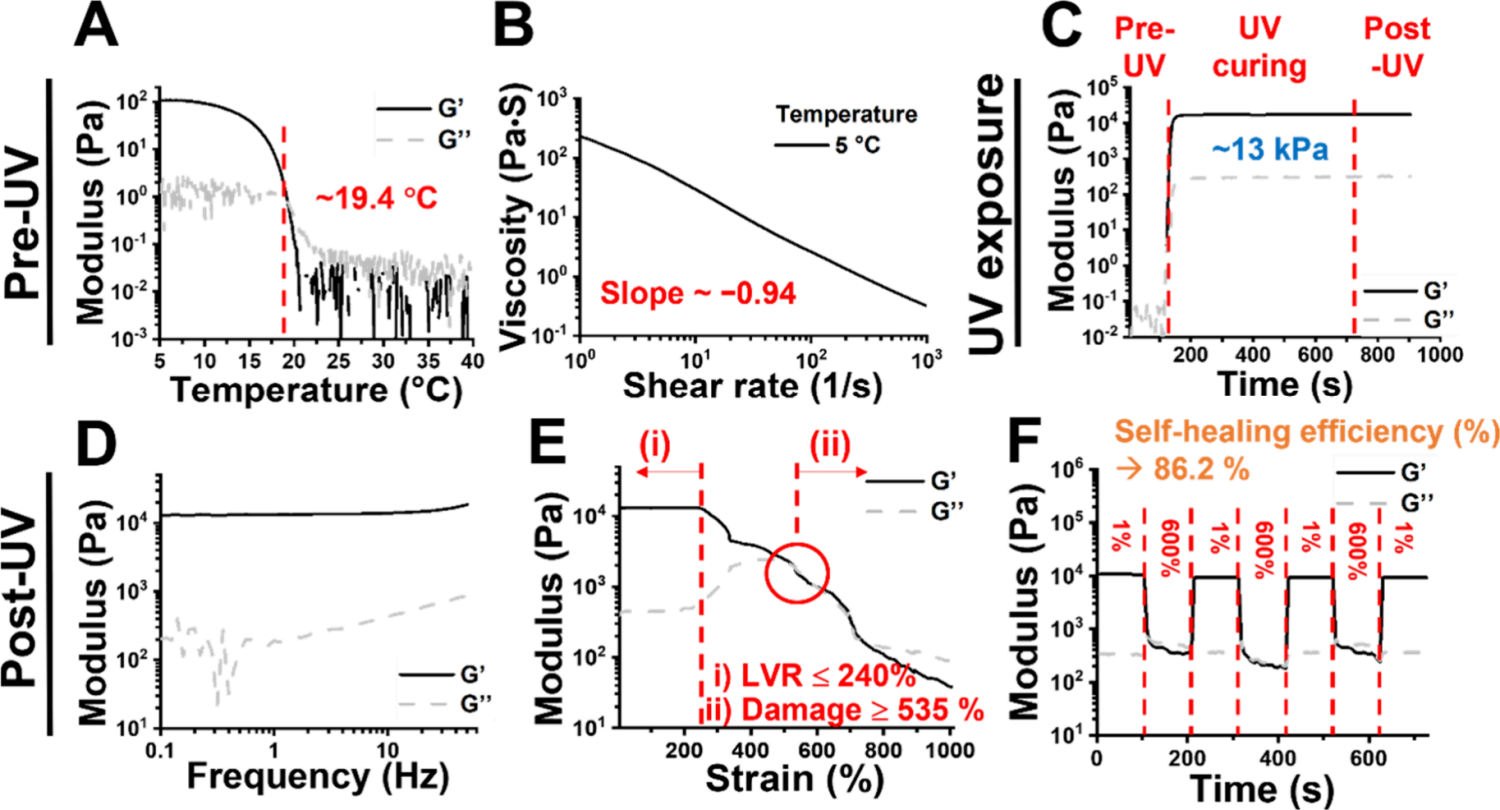
Rheological behavior of PNG′4 precursor and post-UV hydrogel, represented by storage (*G*′) and loss (*G*″) moduli. (A) The temperature sweep for PNG′4 precursor was performed between 5 and 40 °C, with measurements taken at 1 Hz and 1% strain. (B) Measurement of steady shear viscosity for gel-like precursor was measured at 5 °C across a shear rate range from 0.3 s^–1^ to 1000 s^–1^. (C) The time sweep experiment was performed at 25 °C, 1 Hz frequency, and 1% strain to capture the rheological evolution induced by UV exposure. (D) Frequency-dependent rheological behavior of the cured PNG′4 hydrogel was conducted at 25 °C and 1% strain over a frequency range of 0.1–50 Hz. (E) The strain sweep experiment of cured PNG′4 hydrogel was performed at 25 °C and 1 Hz, with dynamic strains ranging from 0.1% to 1000%. The critical damage strain was observed at 535%. Notably, a LVR region was observed up to 240% strain. Besides, *G*′ remained close to *G*″ in the strain range of 535% to 740%. (F) The damaging-healing cycle was performed at 1 Hz, with oscillatory strain continuously alternating between 1% and 600%.

Self-healing performance of the PNG hydrogels was investigated through a damage-healing cycle experiment. Among the four selected PNG hydrogels, PNG′4 hydrogel showed significant self-healing ability (with an efficiency of ∼87%, as shown in Figure 3F). PNG′4 with an optimal rheological performance and higher self-healing efficiency was selected for the subsequent experiments (Table S2). In the meantime, the suboptimal PNG3, PNG′3, and PNG′5 hydrogels also showed a broad LVR region within the strain range up to 130%, 250%, and 150% respectively (Figure S3Ai,Bi,Ci). The damage strain of the PNG3, PNG′3, and PNG′5 hydrogels was about 320%, 560%, and 520% respectively, as illustrated in Figure S3Aii,Bii,Cii. These hydrogels showed less effective self-healing as compared to PNG′4 (Table S2). The rheological properties of post-UV pure 20 wt % PNIPAM hydrogel and 4 wt % GelMA hydrogel (Figure S4) are also listed in the table for comparison.

### 3.4 SAXS Analyses of PNG Hydrogel

Figure 4A–D illustrates the one-dimensional SAXS curves collected from the PNG′4 precursor and hydrogel, in comparison to pure PNIPAM hydrogel and GelMA hydrogel. The q values between 0.002 and 0.02 Å^–1^ were categorized as the low-q region, whereas those from 0.02 Å^–1^ to 0.2 Å^–1^ were considered the high-q region. As shown in Figure 4A, the pure 20 wt % PNIPAM hydrogel initially exhibited a slight decrease, followed by a subsequent increase in scattering intensity within the low-q region as the temperature rose, indicating the shrinkage and compaction of the PNIPAM cross-linked gel structure. In the high-q region, PNIPAM chains started to form the intramolecular hydrogen bonding that led to structural heterogeneity and aggregation. Figure 4B presents the SAXS patterns of the 4 wt % GelMA hydrogel measured across different temperatures. The hump intensity at 0.03 Å^–1^ increased slightly with temperature, which indicated a decrease in polymer chain scale due to the helix–coil transition. Meanwhile, the curves for the PNG′4 precursor solution are presented in Figure 4C. Heating the PNG′4 precursor solution led to an increase in scattering intensity within the low-q region, accompanied by the emergence of a shoulder at 0.006 Å^–1^ near 40 °C. Figure 4D shows that heating the post-UV PNG′4 hydrogel led to an intensity increase and the appearance of two shoulders at 0.003 Å^–1^ and 0.006 Å^–1^ in the low-q range. The appearance of the shoulder at 0.003 Å^–1^ (compared to the PNG′4 precursor) also suggested a newly formed, larger structure ranging from 200.3 to 104.1 nm after photo-cross-linking.

**Figure 4:**
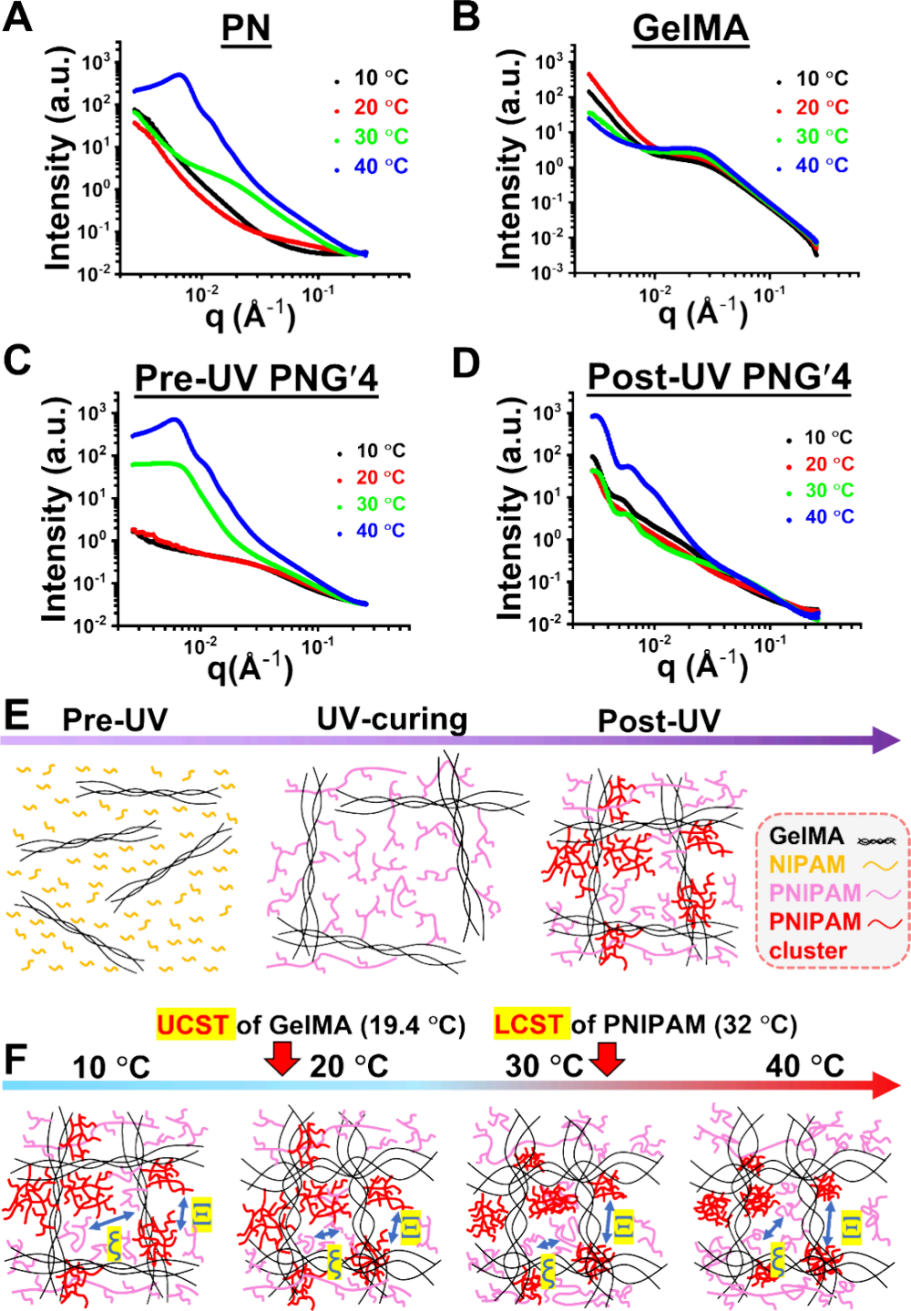
SAXS characterization of PNIPAM (PN), GelMA hydrogels, and the PNG′4 precursor solution and hydrogel, with proposed structural evolution of PNG′4 hydrogel. (A) SAXS patterns of the pure 20 wt % PNIPAM hydrogel were obtained during a stepwise heating from 10 to 40 °C. (B) SAXS patterns were collected for the 4 wt % GelMA hydrogel over the temperature range of 10 to 40 °C. (C) SAXS patterns of PNG′4 precursor obtained at various temperatures. (D) SAXS patterns of PNG′4 hydrogel were recorded under different temperature conditions. The PNG′4 hydrogel was prepared through UV-induced cross-linking at 4 °C. (E) Structural changes of hydrogel before and after photo-cross-linking at 4 °C, as the PNIPAM side chain randomly forms with clustering structure after photo-cross-linking. (F) The structure of post-UV PNG′4 hydrogel under different temperatures. During heating from 10 to 40 °C, the static correlation length (Ξ, between the PNIPAM clusters) of the hydrogel network gradually increases while the dynamic correlation length (ξ, between GelMA or PNIPAM) decreases sharply between 10 and 20 °C.

The scattering profiles were fitted with eq 4–9, and the fitting curves are shown in Figure S5. The fitting model includes a sphere model (eq 5) coupled with a hard-sphere interaction model (eq 6) to describe the PNIPAM clusters and their correlation, and the Ornstein–Zernike equation (eq 8) to describe the thermal fluctuations of the structural lengths ξ and Ξ. Excellent consistency between the fitted results and the experimental SAXS profiles was observed throughout the thermal treatment. Table 2 summarizes the key parameters extracted during the heating process, including the cluster radius, the correlation length between the clusters, and the correlation length between GelMA and PNIPAM chains. The cluster radius of PNG′4 decreases steadily over the temperature range of 10 to 40 °C, except for that at 20 °C. The static correlation length, which indicates the average correlation length between the PNIPAM clusters, increases progressively with rising temperature. The dynamic correlation length, which indicates the average correlation length between GelMA or PNIPAM chains, decreases as the temperature rises from 10 to 30 °C but increases thereafter at the temperature 40 °C. Figure 4E,F proposes the cross-linked structure of PNG′4 hydrogel based on SAXS data and fitting parameters, along with the possible structural changes induced by temperature variations. Figure 4E illustrates the structural changes of PNG′4 precursor at 4 °C under UV irradiation. Before photo-cross-linking, GelMA chains and NIPAM monomers were randomly distributed. During UV exposure, GelMA chains formed covalent bonds while NIPAM monomers polymerized into PNIPAM and partially grafted onto GelMA as PNIPAM side chains. Immediately post-UV, a covalent cross-linked network developed among GelMA chains while the possible interlocking of PNIPAM side chains contributed to the formation of PNIPAM clusters (initial size ∼210 nm). Figure 4F depicts the structural evolution of the UV-cured PNG′4 hydrogel during heating from 10 to 40 °C. At 20 °C, the linear-coils of GelMA can partially unravel as the temperature rises above the UCST of GelMA, leading to smaller clusters (∼140 nm). Beyond 30 °C, the LCST of PNIPAM induces significant contraction of both PNIPAM side chain and clusters (final cluster size ∼167 nm).

**Table 2:** Parameters Obtained by Model Fitting for PNG′4 Hydrogel at Different Temperatures Ranging from 10 to 40 °C

group	cluster radius (nm)	static correlation length Ξ (nm)	dynamic correlation length ξ (nm)
PNG′4 (10 °C)	107.85 ± 2.95	8.11 ± 0.82	13.85 ± 0.37
PNG′4 (20 °C)	71.16 ± 9.18	11.44 ± 0.64	3.95 ± 0.18
PNG′4 (30 °C)	93.98 ± 3.78	13.22 ± 1.36	2.81 ± 0.04
PNG′4 (40 °C)	83.84 ± 1.14	15.72 ± 0.53	5.33 ± 0.46

### 3.5 Needle Injectability of UV-Cured 3D-Printed PNG Hydrogel

Bulk PNG hydrogel can be fabricated from the precursor using a 3D bioprinter. The printing resolution of PNG′4 hydrogel is displayed in Figure 5A. Filaments printed with the 80 μm nozzle had an average diameter of ∼100 μm, while those printed with 210 μm nozzle exhibited an average diameter of ∼260 μm.

**Figure 5:**
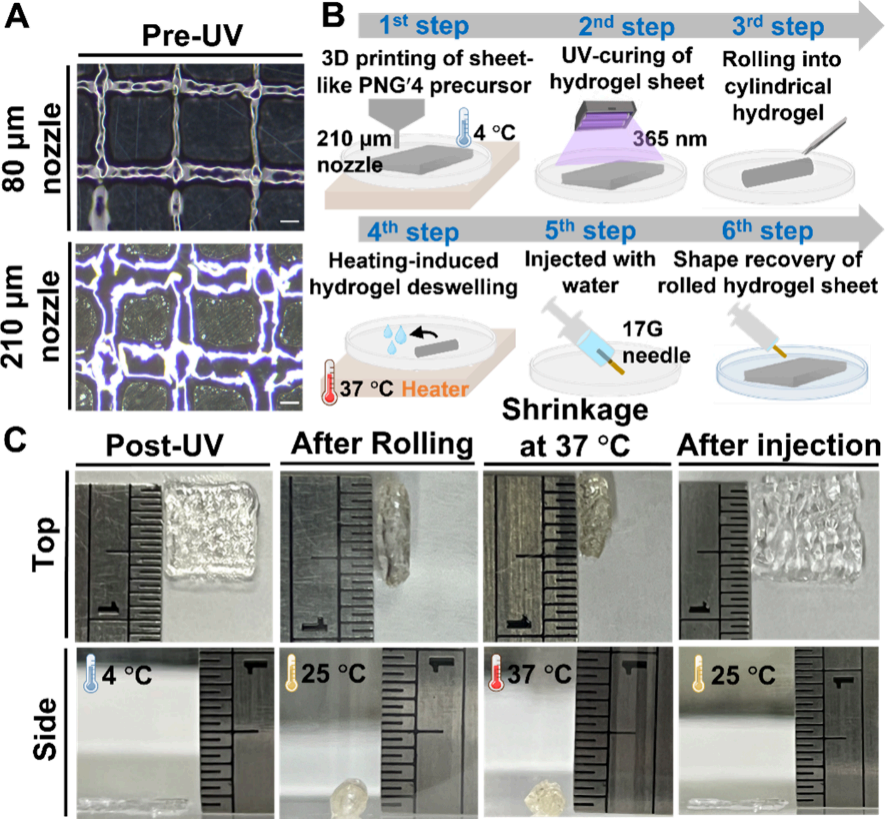
3D printability, needle injectability, and shape recovery of PNG hydrogel. (A) Microscopic views of filaments formed by printing the PNG′4 precursor through an 80 μm/or 210 μm nozzle (scale bar = 200 μm). (B) Schematic diagrams depicting the 3D printing procedure for fabricating the PNG′4 hydrogel sheet and the injectability of the UV-cured PNG′4 hydrogel sheet. (C) Top and side views for the rolled hydrogel sheet under a mild heating condition (37 °C air), and for the recovered sheet structure after injection through a 17G syringe needle at room temperature.

Other than 3D printing, the UV-cured 3D-printed PNG hydrogel also has the capacity of needle injectability and shape recovery. To demonstrate this, a piece of hydrogel was printed as shown in Figure 5B. The PNG′4 precursor was printed using the 210 μm nozzle into a sheet-like structure and achieved a thickness of ∼0.5 mm after stacking two layers at 4 °C. The printed structure was solidified by UV irradiation for 10 min into a hydrogel sheet. The hydrogel sheet was then rolled into a cylindrical hydrogel at 25 °C and placed in 37 °C air for 5 min. The cylindrical hydrogel was then positioned inside a syringe and manually extruded with water through a 17G (1.067 mm) needle. The injected hydrogel restored its original sheet shape at 25 °C after injection. The top and side views of the UV-cross-linked sheet hydrogel are presented in Figure 5C. At 37 °C, the cylindrical hydrogel shrank to ∼55% of its initial volume. After injection, the final sheet hydrogel regained ∼115% of its original volume.

### 3.6 Preparation and Characterization of 3D-Printed PNG/GelMA Bilayer Hydrogel Actuator with Needle Injectability and Bidirectional Actuation

Figure 6 **(A, B)** presents the preparation process of the PNG/GelMA bilayer hydrogel and actuation performance triggered by temperature variations. To optimize the GelMA content applied to the passive layer, the bending angle variations of the bilayer hydrogel at 25 and 37 °C were evaluated (Table S3). The calculation method for the bending angle is shown in Figure 6A. The passive layer made of 7.5 wt % GelMA exhibited the largest bending angle change. For passive layers made of 5 and 10 wt % GelMA, the bending angle changes were slightly smaller. Particularly, all three bilayer hydrogels exhibited bidirectional actuation, i.e., positive bending angles at 37 °C and negative bending angles at 25 °C. Optimal actuation was achieved in the configuration where the active layer consisted of PNG′4 and the passive layer was prepared using 7.5 wt % GelMA. This optimized bilayer hydrogel actuator yielded bending angles of ∼420° at 37 °C and –340° at 25 °C, and was subsequently subjected to further testing.

**Figure 6:**
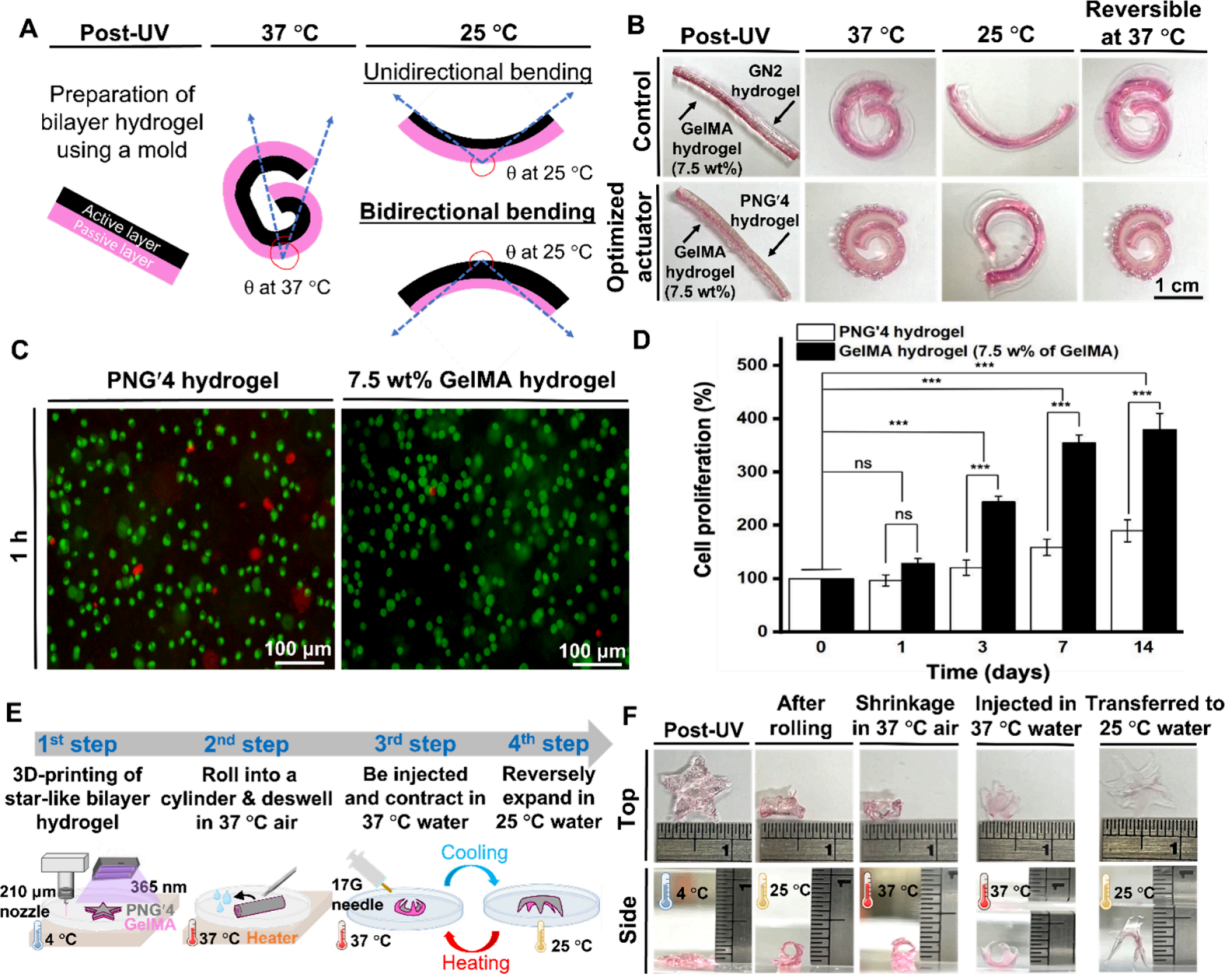
Shape deformation, cytocompatibility, needle injectability, and bidirectional actuation of the bilayer hydrogel actuators. To facilitate visualization of deformation, the passive layer in all bilayer hydrogels was stained with Safranin-O for visualization (scale bar = 1 cm). (A) Schematic illustration for measuring the bending angle of a bilayer hydrogel strip prepared by casting. (B) The new bilayer hydrogel actuator and the control actuator (referenced in Table S3) after photo-cross-linking remained straight at 25 °C. In the cyclic bending test between 25 and 37 °C water, the control actuator exhibited a “6-smile-6” shape transformation, which indicated unidirectional bending. In contrast, the newly developed PNG′4/GelMA bilayer hydrogel actuator showed a “6–9–6”shape transformation, demonstrating bidirectional bending toward the passive layer while reswelling at 25 °C water. (C) The fluorescence microscopic images showing the live/dead staining of C2C12 cells embedded in PNG′4 or GelMA hydrogel for 1 h. Live cells: green; dead cells: red. (D) The long-term proliferation for C2C12 cells embedded in PNG′4 or GelMA hydrogel, determined by the CCK-8 assay. The value of cell proliferation (%) was based on the optical density deducting the blank group (i.e., hydrogels without cells) and then normalized to the value at day 0 of each hydrogel. *** represents *p* < 0.001. (E) Illustration depicting the manufacturing procedure, needle injectability, and reversible actuation of the star-like bilayer hydrogel actuator. (F) Top and side views of the rolled bilayer hydrogel actuator under a mild heating condition (37 °C air). The 3D-printed bilayer hydrogel actuator originally in the shape of a star and later in the shrank roll form was injectable through a 17G syringe needle, contracted into a smaller flower-like structure after injection in 37 °C water, and expanded reversely into a larger spider-like form in 25 °C water. This reversible transformation between the flower and spider morphologies could be repeatedly achieved by alternately immersing the actuator in 37 and 25 °C water.

To evaluate reversibility, the bilayer hydrogel actuator underwent five thermal transitions alternating between 25 and 37 °C water. The bending angles measured across all five cycles are summarized in Table S4. Bending angles remained similar for the rest of the cycles. The actuation images are presented in Figure 6B. The comparison group was a bilayer hydrogel actuator reported in prior literature (GN2/GelMA).^11^ Upon exposure to a 37 °C environment, the newly developed bilayer structure exhibited inward bending directed toward the active layer, accompanied by whitening of the active layer as a result of the phase transition of PNIPAM. The new actuator curved toward the passive layer when transferred back to 25 °C water, i.e., bidirectional, while the comparison group remained unidirectional. The bilayer hydrogel actuator was subjected to progressive temperature changes, and its bending behavior and response time were recorded to characterize the thermally induced actuation (Figure S6). All tests were conducted with fully hydrated samples pre-equilibrated in water at 25 °C until the shape stabilized. Upon immersion in water at 25 °C, the actuator exhibited an initial negative bending angle. As the temperature gradually increased from 25 to 40 °C, the bending angle increased and reversed from negative to positive at approximately 32 °C. An opposite trend was observed during stepwise cooling, with the bending angle decreasing and returning to a negative value below 32 °C. In addition, the actuator was immersed in PBS solution and subjected to five thermal cycles. The bending angle remained consistent across all cycles, indicating stable actuation behavior. Compared to the results obtained in water, the bending amplitude in PBS solution showed a more pronounced decrease at 37 °C and a slight reduction at 25 °C (Table S5). Extended testing further confirmed that the actuator preserved reversible bidirectional bending after 50 cycles in PBS, with only minor amplitude loss relative to the first cycle (Figure S7).

Figure S8A schematically describes the preparation process and temperature-dependent bidirectional actuation behavior of star-like bilayer hydrogel of two different lateral sizes. GelMA hydrogel (7.5 wt %) was first extruded through the 210 μm nozzle to form the passive layer. The PNG′4 precursor was then printed as the active layer. The printed construct was UV-cross-linked for 10 min to form a bilayer hydrogel actuator. The star-like bilayer hydrogel exhibited bidirectional actuation capacity between 37 and 25 °C water. Figure S8B shows a star-like bilayer hydrogel actuator fabricated using 3D printing (nozzle size ∼210 μm). Upon exposure to 37 °C water, the arms of the star-like hydrogel folded inward, forming a flower-like structure. After being transferred to 25 °C water, the actuator reversed its bending direction, forming a spider-like structure. To further demonstrate the capability of printing more sophisticated geometries, a leaf-like bilayer hydrogel actuator was fabricated and evaluated separately (Figure S9). The construct preserved its fine printed features and showed reversible bidirectional bending between 25 and 37 °C. Additionally, strip-shaped bilayer hydrogels were fabricated by 3D printing along either the *X*-axis or *Y*-axis using a 210 μm nozzle and compared with samples prepared by molding. Top-view micrographs of UV-cross-linked strips showed similar surface porosity for *X*-axis and *Y*-axis prints and clear layer alignment parallel to the respective printing direction. All samples were tested in water at 25 and 37 °C. At 25 °C, the *X*-axis printed sample exhibited a smaller bending angle than both the *Y*-axis printed and the molded samples, as shown in Figure S10. A strip-shaped bilayer hydrogel of similar overall dimensions was also printed with an 80 μm nozzle and showed smaller bending angles than the strip printed with a 210 μm nozzle at both 25 and 37 °C (Figure S11).

The cytocompatibility of post-UV cell-laden PNG′4 hydrogel was assessed using live/dead cell staining of myoblast cell line C2C12 (Figure 6C). Compared to the control group, the PNG′4 hydrogel showed a slightly higher proportion of dead cells; however, most cells remained viable, indicating proper cytocompatibility. Cells embedded in both hydrogels exhibited long-term proliferation for 14 days (Figure 6D). Although cells in GelMA hydrogel showed significant growth reaching 382% after 14 days, those in PNG′4 hydrogel also exhibited 190% cell proliferation in 14 days. These findings indicate the cytocompatibility of PNG′4.

The fabrication and actuation strategy of the 3D-printed PNG/GelMA bilayer hydrogel is shown in Figure 6E,F. To demonstrate needle injectability and bidirectional actuation, a smaller star-like hydrogel was printed, as illustrated in Figure 6E. GelMA hydrogel (7.5 wt %) was extruded through the 210 μm nozzle to form the passive layer and the PNG′4 precursor was printed as the active layer. The printed construct was UV-cross-linked to form the bilayer actuator. The star-like hydrogel had a thickness of ∼0.5 mm. Each of the passive and active hydrogel was deposited as a single stacking layer, achieving a thickness of ∼0.25 mm. The actuator was then rolled into a cylindrical shape and placed in 37 °C air for 5 min. It was then loaded into the syringe and injected through the 17G (1.067 mm) needle into 37 °C water. Upon injection, the hydrogel contracted into a flower-like structure and expanded reversely into a spider-like form while transferred to 25 °C water. Figure 6F presents both the top and lateral perspectives of the star-like hydrogel actuator following 3D printing and subsequent UV irradiation. The cylindrical hydrogel contracted to ∼82% relative to its original volume. Upon sequential immersion in water at 37 °C and subsequently at 25 °C, the active layer of the bilayer hydrogel exhibited cyclic deswelling and reswelling behavior driven by thermal fluctuations. The star-like hydrogel exhibits biomimetic actuation resembling morphological adaptations observed in diverse biological organisms.

### 3.7 Bidirectional Actuation of the Self-Healed 3D-Printed Hydrogel Actuator

To construct a bilayer hydrogel with self-healing functionality, PUGG hydrogel was selected as the passive layer because of its intrinsic healing ability. Figure S12A provides an overview of the fabrication process, self-healing evaluation, and thermally driven bidirectional actuation behavior of the PNG/PUGG bilayer hydrogel, serving as a visual guide for the experimental workflow described below. First, a strip-shaped bilayer hydrogel was prepared using a mold and subsequently cut into two pieces. The two segments were brought into contact and allowed to self-heal for 20 min. After healing, the hydrogel strip could be lifted with tweezers without breaking at the cut site, confirming effective self-healability, as shown in Figure S12B. To further assess the self-healing capability under physiologically relevant conditions, additional healing experiments were conducted at 37 °C. Successful reconnection of the cut hydrogel segments was observed under this condition as well (Figure S13). To assess actuation performance after repair, the cut/self-healed bilayer hydrogel was compared with the original/intact bilayer hydrogel. As presented in Figure S12C, both groups bent toward the active layer when immersed in water at 37 °C, although the bending angle of the cut/self-healed group was slightly smaller than that of the original/intact one. Upon transfer to 25 °C water, both groups exhibited reverse bending behavior, while the cut/self-healed group showed a reduction in bending angle. Specifically, the self-healed bilayer hydrogel achieved an angle of ∼380° at 37 °C and ∼−270° at 25 °C. To evaluate reversibility, both hydrogels were subjected to five cycles between 25 and 37 °C water. Table S6 summarizes the bending angles measured over five consecutive cycles, which exhibited similar results across all cycles.

Unlike the previously mentioned mold-fabricated samples, a star-like PNG/PUGG bilayer hydrogel actuator was created using extrusion-based 3D printing. Figure 7A illustrates the fabrication steps, self-healing assessment, and bidirectional actuation performance of the 3D-printed hydrogel actuator, which is further elaborated below. The star-like bilayer actuator was fabricated by sequentially extruding the PUGG precursor and PNG′4 ink through a 210 μm nozzle and depositing them layer by layer to form 12 stacking layers. The printed structure was then exposed to UV light for 10 min to complete polymerization. The final hydrogel actuator had a total thickness of ∼2.5 mm, with the active and passive hydrogel components each comprising six stacking layers to form ∼1.25 mm thickness per component. To verify the self-healing ability, one arm of the star-like hydrogel actuator was cut and then reattached for 20 min. As shown in Figure 7B, the healed hydrogel actuator could be lifted with tweezers without separating at the previous cut site, indicating successful self-healing. Figure 7C presents a comparison of actuation behavior between the self-healed and original hydrogel actuators. Both actuators displayed reversible bending in response to temperature changes; however, the healed hydrogel actuator showed reduced bending amplitude at the healing site.

**Figure 7:**
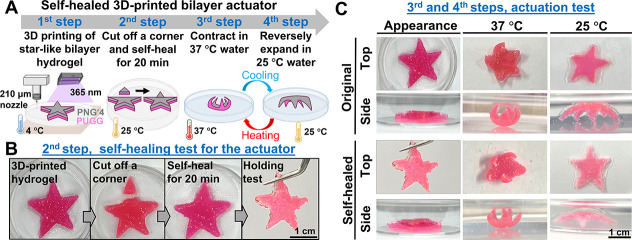
Self-healable bilayer hydrogel actuator was 3D-printed and evaluated for the actuation performance. The actuator was based on the self-healable PNG′4 active layer and PUGG passive layer.^31^ For enhanced contrast and visualization, the passive layer in each actuator was selectively stained with Safranin-O (scale bar = 1 cm). (A) Schematics for the fabrication process, self-healing, and reversible actuation of the star-like bilayer hydrogel actuator. (B) The self-healing behavior of the 3D-printed star-like bilayer hydrogel actuator was observed by cutting off a corner of the actuator and allowing it to self-heal for 20 min. After the actuator heals, it remains intact when picked up with tweezers. (C) Top and side views for the actuation of the 3D-printed star-like bilayer hydrogel actuators are presented. Upon immersion in water at 37 °C, both actuators folded into a compact, flower-like morphology, while reverting to a spider-like configuration at 25 °C.

## 4 Discussion

Responsive hydrogels often suffer from cracking after repeated swelling/deswelling cycles,^33^ while imparting self-healing properties can enhance their lifespan.^34^ The PNIPAM-GelMA (DS 95%) hydrogel reported in a prior study did not have self-healing properties and was somewhat brittle.^11^ In the current study, the PNIPAM-GelMA (DS 47%) (PNG) hydrogels made from different contents of PNIPAM and GelMA reveal the unique self-healing properties and broad linear viscoelastic region (LVR), distinct from the previously reported GelMA (DS 95%)-PNIPAM hydrogel. The amide and carbonyl groups on PNIPAM can form dynamic bonds with the hydroxyl and amine groups of GelMA (Figure 1), which may lead to hydrogen bonding in the PNG network and contribute to self-healing properties. PNG hydrogels using GelMA (DS 47%) provide more of these bonding sites and thus yield higher self-healing efficiency than those using GelMA (DS 95%) under the same solid content. Consistent with this interpretation, IR spectra support hydrogen bonding formation in the cross-linked network. After photo-cross-linking, the N–H/O–H stretching band at 3400–3200 cm^–1^ intensified and the C = O (∼1660 to ∼1645 cm^–1^) and amide II (∼1545 to ∼1535 cm^–1^) bands shifted to lower wavenumbers. These spectral changes are characteristic of hydrogen bonding and provide direct evidence (Figure S1). The self-healing ability of PNG hydrogels may enhance structural durability and offer resistance to prolonged contraction-induced deformation.

PNIPAM-based hydrogels are commonly synthesized via ammonium persulfate (APS)-initiated free radical polymerization or photopolymerization. The APS route generally offers a narrow processing window and is not compatible with extrusion-based 3D printing, whereas photopolymerization has enabled PNIPAM hydrogels to be applied in 3D printing as demonstrated in previous reports.^35^ In this study, we further advanced this concept by incorporating GelMA as a printable matrix and employing VA-086 as a more biocompatible initiator. This adjustment improved precursor moldability and enabled extrusion-based 3D printing. It also enhanced cytocompatibility. As a result, we obtained a hydrogel system suitable for the development of bioactuators. Here in this study, we employed UV-initiated photopolymerization to form PNIPAM hydrogels, providing an extended working window that enables customizable shaping. Using this approach, a self-healing hydrogel network was successfully fabricated from 20 wt % pure PNIPAM (Figure S4); however, the PNIPAM hydrogel exhibits low moldability. The addition of a small amount of GelMA to the PNIPAM hydrogel system significantly enhances its moldability and imparts injectability. To ensure the properties such as the injectability of the PNG precursor at 4 °C and the self-healing ability of the post-UV hydrogel (Figure 2B,C), the formulation was optimized by varying the NIPAM-to-GelMA ratio and the GelMA substitution degree (Table S1). The total solid content of PNG hydrogel was fixed at 20 wt % in order to rapidly screen out the ratio with the aforementioned properties by changing the NIPAM-to-GelMA ratio in the system. Previous literature revealed that GelMA-based precursors with GelMA concentration over 3 wt % had better flow property at low temperature.^36^ Indeed, we observed that PNG precursors with GelMA contents below this threshold showed poor shear-thinning behavior, whereas post-UV PNG hydrogels with GelMA contents exceeding ∼3 wt % (DS 95%) began to lose the self-healing capacity likely due to reduced entanglement of shorter PNIPAM side chains. For formulas with DS 47% GelMA, those containing 3–5 wt % GelMA owned sufficient self-healability. This is probably associated with better packed PNIPAM chains through more abundant hydrogen bonding. The PNG′4 group was selected as the optimized formulation owing to its desirable properties, including the formation of an injectable physical gel from its precursor at 4 °C, the healing of minced post-UV hydrogel pieces at 25 °C, and shrinkage to ∼55% of its original size at 37 °C. During the optimization process, a concurrent decrease in swelling ratio and gel fraction was also observed in formulations containing DS 47% GelMA (Table 1). This phenomenon may be attributed to the increased presence of hydrophilic groups, which tend to form hydrogen bonds and generate compact amorphous regions. These physically dense domains are formed through hydrogen bonding between hydrophilic groups that are already associated with water molecules and therefore restrict further expansion of the network during swelling, despite a lower chemical cross-linking density. This suggests that the swelling behavior of PNG hydrogels is influenced not only by covalent cross-links but also by physical interactions within the network.

Hydrogels with 3D printability enable the fabrication of complex and customizable architectures, which are essential for applications requiring precise structural control.^37,38^ According to literature, the more negative slope of the linear fit of viscosity against shear rate in a double-logarithmic scale indicates better printability.^39^ The PNG precursor solution exhibited excellent shear-thinning properties at 5 °C (Table S2), allowing for extrusion and 3D printing. After photo-cross-linking, the storage modulus of the PNG′4 hydrogel (13 kPa) is an order higher than that of pure 20 wt % PNIPAM (1.2 kPa) and 4 wt % GelMA (0.2 kPa), likely due to the formation of a double-network structure composed of covalent GelMA cross-links and PNIPAM side chains. The strain sweep experiment indicated that PNG′4 hydrogel showed a broad LVR for strain up to ∼240% (Figure 3Ei). A recent study has reported the formation of PNIPAM brushes after PNIPAM polymerization.^40^ The broad LVR observed in the present study is probably associated with the interlocked PNIPAM network, where reversible “sacrificial bonds” (such as hydrogen bonding, originating from the extensively entangled PNIPAM side chains) break and reform to dissipate stress, yielding a tough hydrogel capable of undergoing significant deformation and recover its structure.^41,42^ In this work, we use the term “toughness” only in a qualified sense to indicate rheology-inferred damage tolerance under oscillatory shear rather than fracture toughness measured by crack-resistance tests. Studies have shown that covalently cross-linked double-network structures, formed by embedding physically cross-linked networks rich in hydrogen bonding, exhibit favorable mechanical strength and good self-healing performance.^43,44^ Based on literature precedents, the correlation between the microstructure of PNG′4 hydrogel and its self-healing behavior can be inferred. Upon damage, hydrogen bonds between PNIPAM side chains at the fractured interfaces break rapidly, exposing bonding sites that can reconstruct new hydrogen bonds during self-healing and restore most of the original mechanical modulus. However, the modulus arising from a large number of hydrogen bonds remains lower than that of the original PNG system, and the difficulty of achieving perfect contact at the fractured interface results in a self-healing efficiency of ∼86%.

Understanding the relationship between the microstructure and macroscopic properties of hydrogels is critical for rational material design.^45^ Previous studies have shown that pure GelMA hydrogels (covalently UV-cross-linked) lack self-healing properties and are unable to recover after damage.^46^ Accordingly, GelMA serves as a static three-dimensional covalent network within the PNG hydrogel system. In this study, pure 20 wt % PNIPAM (PN) hydrogel exhibited 100% self-healing capability (Figure S4E), likely resulting from dense PNIPAM chains forming a physically cross-linked network. Therefore, PN acts as a dynamic physical cross-linking network in the PNG system. In contrast, the previously reported GN hydrogel showed no self-healing behavior,^11^ possibly because the formed PNIPAM chains had insufficient density and length, resulting in a scarcity of available dynamic bonds. Notably, the PNG′4 hydrogel exhibited a shoulder in the SAXS profile that was absent in both PN and GelMA hydrogels (Figure 4A,B,D), which is attributed to the heterogeneous aggregation of PNIPAM side chains within the GelMA covalent network. Literature has reported that long side chains interpenetrate to form tight interlocks, and that higher grafting densities and longer side chain lengths can enhance chain mobility.^47^ Based on these findings, it is speculated that during self-healing, PNIPAM side chains reinterpenetrate under the influence of hydrogen bonding and van der Waals forces to reconstruct interlocking structures. To observe the native cluster organization, SAXS measurements were performed in situ under full hydration. SEM and TEM were not used as primary evidence because SEM requires dehydration or freeze-drying and TEM requires dilution and staining, which can distort PNIPAM-rich clusters.

Further validation of these structural assumptions and deeper insight into the temperature-dependent microstructural evolution of the PNG hydrogel were obtained through SAXS analyses conducted over the temperature range of 10 to 40 °C. The shoulder in the UV-cross-linked PNG′4 hydrogel was detected at 10 °C and remained evident up to 40 °C (Figure 4D). Based on the fitting results (Table 2), the structural diagram of PNG′4 hydrogel was proposed (Figure 4F). There is a gradual decrease in cluster radius with rising temperature, attributed to the contraction of PNIPAM side chains, which causes the clusters to become denser. Notably, the cluster radius sharply decreases at 20 °C, possibly because the clustered structure is obscured by the partial unraveling of GelMA linear-coils near the UCST of PNG′4. The steady increase in static correlation length (Ξ) with temperature reflects the enlarged distance between clusters as they shrink. Conversely, dynamic correlation length (ξ) decreases with rising temperature because the unraveling of GelMA linear-coils at 20 °C brings the PNIPAM chains closer together. At 30 °C, the PNIPAM chains begin to bend and contract, further reducing interchain spacing. At 40 °C, the fully contracted PNIPAM chains slightly increase the interchain spacing. The cluster radius decreased substantially from 10 to 40 °C, likely accounting for the thermoresponsive deswelling behavior of the PNG′4 hydrogel. This nanoscale shrinkage is consistent with the macroscopic deswelling behavior, where the hydrogel volume decreased to ∼55% of its original state at 37 °C (Figure S2A). The structural factors and network model obtained by SAXS fitting can justify the macroscopic and mechanical properties of PNG hydrogels.

For environment-responsive hydrogels, achieving sufficient toughness is crucial for applications that require resistance to fracture while preserving structural integrity.^48^ Mechanical robustness is particularly important in minimally invasive delivery, where the hydrogel must endure high shear stress and subsequently recover its original shape.^49^ In this study, post-UV PNG′4 hydrogel demonstrated needle injectability and shape recovery. The rolled PNG′4 hydrogel sheet heated to 37 °C can be injected using fine needles under manually hydraulic pressure (Figure 5B). The needle injectability of the cured PNG′4 hydrogel may be ascribed to its broad LVR and temperature responsiveness, which enable the hydrogel to be compressed and deform under stress. The shape recovery after injection was driven by the elastic recovery of the PNG hydrogel. Within LVR, the elasticity of cross-linked network allowed PNG hydrogel restored its original shape. This broad LVR is a key feature that may account for its ability to pass through a syringe needle without fragmentation and to preserve the integrity of the hydrogel after needle injection. Such shape recoverable hydrogels may serve as injectable scaffolds to support cell growth while mimicking the flexible and malleable properties of natural tissues.

Stimulus-responsive hydrogels with biocompatibility are similar to extracellular matrices in terms of structure and physicochemical properties and have recently attracted significant research interest for bioactuator fabrication.^50^ Emerging tough hydrogels allow repeated large-strain deformations during cyclic actuation, making them suitable for bioactuators and biomedical applications.^51^ Bidirectional hydrogel actuators enable more complex motion behaviors than unidirectional actuators without requiring multiple stimuli responsiveness.^52,53^ In this study, we employed a tough PNG hydrogel with thermoresponsive properties as the active layer of the bilayer hydrogel actuator. Compared to the unidirectional actuator similarly from PNIPAM and GelMA,^11^ the new PNG/GelMA actuator exhibits highly reversible bidirectional bending, associated with the unique active layer composition. With an optimized passive layer (7.5 wt % GelMA), the actuator results in the greatest bending angle variation, reaching 420° at 37 °C and −340° at 25 °C (Table S4). The bidirectional actuation behavior arises from internal stress mismatch between the PNIPAM-rich active layer and the GelMA-rich passive layer. Heating above the LCST stores elastic strain as PNIPAM clusters collapse, producing bending toward the active side. Cooling rehydrates the network and releases the stored strain to generate reverse bending with the aid of bulk reswelling. Recent hydrogel studies support this stress–recoil mechanism, where reversible actuation is driven by elastic energy storage and release.^54^ Importantly, all actuation tests were conducted with fully hydrated samples pre-equilibrated in water at 25 °C, ensuring that the negative bending angle originates from elastic strain release and reswelling during cooling rather than initial swelling. The advanced performance of the new PNG/GelMA bilayer actuator is further demonstrated by its needle injectability and actuation retention of actuating capacity after injection (Figure 6F). However, cell viability during injection was not assessed because this study aimed to verify postinjection structural integrity and retention of actuation. Future work will quantify the viability and functionality of PNG-encapsulated cells during and after injection to evaluate suitability as a cell delivery vehicle.

To better simulate physiological temperature dynamics, progressive temperature ramps were applied (Figure S6). Upon immersion in water at 25 °C, the actuator initially adopted a negative bending angle. This observation likely stems from differential swelling between the active PNIPAM layer and the passive layer, which induces a prebend even before temperature increase. Moreover, the actuation tests were conducted in PBS solution to approximate the ionic conditions present in physiological fluids (Table S5). A reduction in bending amplitude was observed compared to water at both 25 and 37 °C. This attenuation may result from competitive hydrogen bonding, where ions in PBS interfere with the interactions between water molecules and the polar functional groups of the hydrogel. Such interference may reduce the swelling capacity of the hydrogel by limiting the formation of hydrogen bonds within the network, thereby suppressing the volume change in the PNIPAM-based active layer and diminishing the extent of actuation. Despite this attenuation, extended fatigue experiments showed that the actuator preserved bidirectional bending after 50 cycles in PBS, highlighting robustness under physiologically relevant conditions (Figure S7). To investigate the influence of printing-induced directionality on actuation behavior, bilayer hydrogel strips were fabricated along different printing paths and compared with molded samples (Figure S10). The smaller bending angle observed in the *X*-axis printed sample may result from direction-dependent mechanical resistance during reverse bending. Such resistance is possibly caused by anisotropic microstructures formed along the printing path during layer-by-layer deposition, as observed by top-view optical microscopy. In line with this mechanism, strips printed with an 80 μm nozzle showed smaller bending angles than 210 μm prints because finer filaments formed denser and more uniform layers. The resulting increase of in-plane constraint limited the extension and compression during bending and reduced the bending angle. In addition, the maximum printable thickness was restricted by the need to maintain PNG hydrogel at 4 °C during deposition. In the open-frame setup, cooling efficiency decayed with increasing stack height, thereby limiting the achievable height of multilayer constructs. Moreover, long-term cell proliferation was observed in both layers of the bilayer hydrogel actuator and indicated a favorable microenvironment for cell growth while degradation studies confirmed that both layers were degradable (Figure S14). The 7.5 wt % GelMA group serves as a positive control to contextualize PNG′4 performance. PNG′4 shows a lower proliferation rate because it contains 4 wt % GelMA and therefore provides fewer adhesion motifs. Near 37 °C the PNIPAM network collapses, which tightens the mesh and increases local hydrophobicity; this can limit cell spreading and transport. Although proliferation is lower than in GelMA, raising the GelMA fraction or adding adhesive cues such as RGD may enhance proliferation while preserving actuation performance. This soft yet tough actuator with bidirectional bending behavior and biocompatibility is expected to be a promising candidate for utilization in minimally invasive delivery and actuation.

Stimuli-responsive hydrogel actuators fabricated from self-healing hydrogels can autonomously repair damage and maintain functionality, thereby extending device lifespan and reliability.^24^ The passive layer of the bilayer hydrogel actuator was replaced with a self-healable PUGG hydrogel, enabling both layers of the actuator to possess self-healing capability. The PNG/PUGG bilayer hydrogel actuator was cut and subjected to the bending test after self-healing (Figure S12 **)**. The cut/self-healed group exhibited a smaller bending angle compared to the original/intact group. The observed decline in actuation performance is attributed to incomplete structural recovery at the self-healing interface, which hinders efficient force transmission and leads to a diminished bending amplitude. The PNG/PUGG bilayer hydrogel actuator was fabricated via 3D printing and then subjected to self-healing tests (Figure 7). The 3D-printed self-healable bilayer hydrogel can form more complex structures and recover most of its properties after mechanical damage. The 3D-printed star-like bilayer hydrogel actuator retains its bidirectional actuation capacity after a corner was cut off and subsequently self-healed. However, the PNG/PUGG bilayer hydrogel actuator exhibited a smaller bidirectional bending angle compared to the PNG/GelMA actuator. This difference is likely due to the higher storage modulus of the PUGG passive layer (*G*′ ∼ 1.2 kPa) relative to the GelMA passive layer (*G*′ ∼ 0.2 kPa), which may impose greater mechanical resistance on the active layer during contraction and reverse bending.^55^ Although the bidirectional bending performance was limited, the integrated experiment demonstrated that all functional capabilities of the PNG hydrogel were preserved, resulting in a self-healable, biocompatible hydrogel actuator with 3D printability and bidirectional actuation capability. This work offers promising prospects for the future development and integration of multifunctional hydrogel actuators.

Overall, this study establishes a straightforward strategy to fabricate a soft yet tough, self-healable PNIPAM-GelMA (PNG) hydrogel as an active layer of the bilayer hydrogel actuator that possesses 3D printability, needle injectability and bidirectional actuating capacity. Rheological studies confirm a dual-network structure formed by PNIPAM side-chain interlocking in PNG hydrogel, contributing to increased modulus and a broad linear viscoelastic region (LVR). SAXS analysis and model fitting was employed to elucidate the cluster-like structure of PNG hydrogel as well as its self-healing behavior and thermoresponsive morphology. The PNG/GelMA bilayer hydrogel exhibits reversible bidirectional bending, possibly driven by the contraction and expansion of PNIPAM clusters. Moreover, the 3D biomimetic actuator retains bidirectional actuation behavior after pushing out of a 17G (1.067 mm) syringe needle into 37 °C water. With the passive GelMA layer replaced by PUGG hydrogel, the 3D-printed PNG/PUGG self-healable bilayer hydrogel actuator still retains its bidirectional actuation capacity after being cut and self-healed. The integration of self-healing, tough, and thermoresponsive properties positions PNG hydrogel and the soft actuator as promising candidates for advanced biomedical applications, including artificial muscles and minimally invasive procedures.

## 5 Conclusion

UV-cross-linked PNIPAM-GelMA (PNG) hydrogels exhibiting self-healing property, mechanical toughness, and biocompatibility were successfully prepared utilizing GelMA (DS 47%) and NIPAM monomer. The optimized PNG hydrogel made from 4 wt % GelMA and 16 wt % NIPAM shows a storage shear modulus of ∼13 kPa and a broad linear viscoelastic region (LVR) extending up to 240% shear strain. These features of elasticity and structural integrity markedly surpass those of pure PNIPAM hydrogel (*G*′ ∼ 1.2 kPa) and pure GelMA hydrogel (*G*′ ∼ 0.2 kPa), suggesting the formation of a robust double-network structure reinforced by interlocked PNIPAM side chains. Rheological studies demonstrated a self-healing efficiency of ∼86%, attributed to dynamic physical cross-linking of PNIPAM side chains that reinterpenetrate and entangle to restore the interlocked network structure. SAXS analysis provides insight into the nanoscale structural dynamics of the hydrogel network after photo-cross-linking and unveils the temperature-dependent morphological transformation associated with PNIPAM cluster formation and contraction. The SAXS model fitting of PNG hydrogel identifies the presence of spherical clusters, of which the radius decreased from 107.85 nm at 10 °C to 83.84 nm at 40 °C, accompanied by variations in static and dynamic correlation lengths. The SAXS analysis and model fitting verify the structure–property relationship of PNG hydrogel, highlighting the significant influence of PNIPAM clusters on chain interactions within the hydrogel network. Furthermore, the PNG/GelMA bilayer hydrogel demonstrates 3D printability and exhibits reversible bidirectional actuation, with bending angles of approximately +420° at 37 °C and –340° at 25 °C, driven by thermoresponsive swelling/deswelling. The 3D-printed hydrogel actuator demonstrates shape recovery after being pushed out of a syringe needle and retains the functionality of bidirectional actuation. To achieve integrated self-healing functionality of the bilayer hydrogel actuator, the passive GelMA layer was replaced with a self-healable PUGG hydrogel, enabling both the active and passive layers to possess self-healing capability. The 3D-printable PNG/PUGG bilayer hydrogel retained the reversible bidirectional actuation after self-healing, achieving bending angles of approximately 380° at 37 °C and –270° at 25 °C following a healing period of 20 min, thereby confirming autonomous damage repair and functional recovery. This multifunctional PNG hydrogel platform, integrating mechanical toughness, self-healing ability, needle injectability, and reversible bidirectional actuation properties, underscores its potential in advanced biomedical applications, including minimally invasive delivery, artificial muscles, and responsive soft actuators for adaptive therapeutic use.

## Supplementary Material



## Notes

Characterization of PNG hydrogels and bilayer hydrogel actuators fabricated therefrom (PNG/GelMA, PNG/PUGG); and data of formulation screening, physicochemical analysis, thermoresponsive actuation, SAXS fitting profiles, degradation (all PNG), and rheological properties (PNG, PNIPAM, and GelMA hydrogels); effects of passive layer, PBS solution, stepwise actuation, multicycle actuation, printing orientation, and resolution (all PNG/GelMA), as well as self-healing (PNG/PUGG) on bending angle; shape deformation of star-like and leaf-like actuators (PNG/GelMA) (PDF)
