# Supplementary material for: A Biocompatible and Self-Healable 3D-Printed Bidirectional Hydrogel Actuator with Needle Injectability
Source: ACS Appl Mater Interfaces. 2025 Oct 2;17(42):58709–24. doi: 10.1021/acsami.5c14232 (PMC12557206; doi:10.1021/acsami.5c14232)
Supplement: Supplementary file 1 [file am5c14232_si_001.pdf]

# Supporting Information

# A biocompatible and self-healable 3D-printed bidirectional hydrogel actuator with needle injectability

*Kai-Ruei Yang, Qian-Pu Cheng, Shan-hui Hsu**

Institute of Polymer Science and Engineering, National Taiwan University,

Taipei 106319, Taiwan, Republic of China

* Corresponding author: Shan-hui Hsu

Institute of Polymer Science and Engineering, National Taiwan University, No. 1,

Sec. 4 Roosevelt Road, Taipei 106319, Taiwan, R.O.C.

orcid.org/0000-0003-3399-055X; Phone: +886-2-3366-5313;

E-mail: shhsu@ntu.edu.tw; Fax: +886-2-3366-5237

**Supplemental tables:6; Table S1 to Table S6**

**Supplemental figures:11; Figure S1 to Figure S14**

**Table S1.** The flow property and self-healing ability of hydrogels prepared from NIPAM and GelMA in different ratios. The flow property was evaluated by the appearance and needle injection at 4 °C before photo-crosslinking (Test 1). The self-healing ability was evaluated by healing of minced pieces at 25 °C after photo-crosslinking (Test 2). Based on the table, groups PNG3, PNG′3, PNG′4, and PNG′5 (passing both tests) were selected for the main study.

| Groups | Composition (wt %) | | | Assessment * | |
|---|---|---|---|---|---|
| | NIPAM | GelMA (DS 95%) | GelMA (DS 47%) | Test 1 | Test 2 |
| PN | 20 | 0 | 0 | Sol state | Healed |
| PNG1 | 19 | 1 | 0 | Sol state | Healed |
| PNG2 | 18 | 2 | 0 | Sol state | Healed |
| PNG3 | 17 | 3 | 0 | Injectable gel | Healed |
| PNG4 | 16 | 4 | 0 | Injectable gel | Cracked |
| PNG5 | 15 | 5 | 0 | Injectable gel | Fragmented |
| PNG10 | 10 | 10 | 0 | Difficult to inject | Fragmented |
| PNG′3 | 17 | 0 | 3 | Injectable gel | Healed |
| PNG′4 | 16 | 0 | 4 | Injectable gel | Healed |
| PNG′5 | 15 | 0 | 5 | Injectable gel | Healed |
| PNG′6 | 14 | 0 | 6 | Injectable gel | Cracked |

*Test 1: Injection test at 4 °C (before UV curing)
Test 2: Self-healing test at 25 °C (after UV curing)

**Table S2.** A summary for the rheological properties of selected PNG hydrogels.

| **Groups** | **Sol-gel transition temperature (°C)** | **Slope of viscosity** | **Storage modulus (kPa)** | **Damage strain (%)** | **Self-healing efficiency (%)** |
|---|---|---|---|---|---|
| PN | N/A | N/A | 1.2 | 160 | 100 |
| GelMA | 28.1 | –0.81763 | 0.2 | 582 | N/A |
| PNG3 | 17.1 | –0.86327 | 18 | 320 | 53.2 |
| PNG′3 | 16.7 | –0.90613 | 8.5 | 560 | 71.5 |
| PNG′4 | 19.4 | –0.94292 | 13 | 535 | 86.2 |
| PNG′5 | 21.1 | –1.01684 | 17 | 517 | 60.9 |

*PN represents photo-crosslinked hydrogel from 20 wt% NIPAM solution, and GelMA represents photo-crosslinked hydrogel from 4 wt% GelMA (DS 47%) solution. The composition of PNG hydrogels: refers to **Table 1**.

**Table S3**. The bending angles in 37 °C and 25 °C water for bilayer hydrogels of which the passive layer was made of GelMA at different concentrations. The formula of the control group was based on the previous literature[1].

| **Groups** | **Deswelling at 37 °C** | **Reswelling at 25 °C** |
|---|---|---|
| | **Bending angle (°)** | **Bending angle (°)** |
| Active layer: PNG′4 hydrogel<br>Passive layer: GelMA hydrogel (5 wt%) | 361.66 ± 5.13 | –332.75 ± 7.76 |
| Active layer: PNG′4 hydrogel<br>Passive layer: GelMA hydrogel (7.5 wt%) | 420.25 ± 9.07 | –347.67 ± 7.13 |
| Active layer: PNG′4 hydrogel<br>Passive layer: GelMA hydrogel (10 wt%) | 386.50 ± 5.29 | –250.66 ± 8.80 |
| Control (refer to the previous literature[1])<br>Active layer (GN2 hydrogel): GelMA DS 95% (2.5 wt%) + NIPAM (7.5 wt%)<br>Passive layer: GelMA hydrogel (7.5 wt%) | 397.33 ± 3.77 | 281.56 ± 6.97 |

**Table S4**. Bending angles of the new bilayer hydrogel actuator with bidirectional actuation during repeated bending in 37 °C and 25 °C water for five cycles. After the second cycle, the hydrogel actuator exhibited similar bending angles as the second cycle.

| Groups | 1st cycle | | 2nd cycle | | 3rd cycle | | 4th cycle | | 5th cycle | |
|---|---|---|---|---|---|---|---|---|---|---|
| | **37 °C** | **25 °C** | **37 °C** | **25 °C** | **37 °C** | **25 °C** | **37 °C** | **25 °C** | **37 °C** | **25 °C** |
| PNG′4/GelMA bilayer actuator | 420 ± 9 | −348 ± 7 | 416 ± 5 | −336 ± 6 | 418 ± 4 | −339 ± 6 | 421 ± 9 | −341 ± 6 | 416 ± 5 | −338 ± 6 |
| Control actuator[11] GN2/GelMA bilayer actuator | 397 ± 4 | 282 ± 7 | 388 ± 7 | 276 ± 5 | 386 ± 5 | 278 ± 7 | 385 ± 5 | 280 ± 7 | 388 ± 7 | 279 ± 5 |

**Table S5.** Bending angles of the bilayer hydrogel actuator during five cycles in PBS solution between 25 °C and 37 °C.

| Group | 1st cycle | | 2nd cycle | | 3rd cycle | | 4th cycle | | 5th cycle | |
|---|---|---|---|---|---|---|---|---|---|---|
| | **37 °C** | **25 °C** | **37 °C** | **25 °C** | **37 °C** | **25 °C** | **37 °C** | **25 °C** | **37 °C** | **25 °C** |
| PNG′4/GelMA bilayer actuator (in PBS solution) | 281 ± 7 | −323 ± 5 | 290 ± 6 | −317 ± 5 | 283 ± 9 | −315 ± 3 | 295 ± 6 | −320 ± 4 | 289 ± 5 | −324 ± 4 |

**Table S6.** The cyclic bending angles of the bilayer hydrogel actuator before and after self-healing.

| Groups | 1st cycle | | 2nd cycle | | 3rd cycle | | 4th cycle | | 5th cycle | |
|---|---|---|---|---|---|---|---|---|---|---|
| | 37 °C | 25 °C | 37 °C | 25 °C | 37 °C | 25 °C | 37 °C | 25 °C | 37 °C | 25 °C |
| Original (intact) PNG′4/PUGG bilayer actuator | 398 ± 3 | –340 ± 9 | 392 ± 7 | –334 ± 6 | 393 ± 5 | –332 ± 6 | 396 ± 4 | –341 ± 7 | 393 ± 6 | –338 ± 8 |
| Cut and self-healed PNG′4/PUGG bilayer actuator | 381 ± 6 | –273 ± 4 | 378 ± 5 | –267 ± 6 | 376 ± 4 | –269 ± 8 | 380 ± 4 | –264 ± 9 | 374 ± 7 | –272 ± 6 |

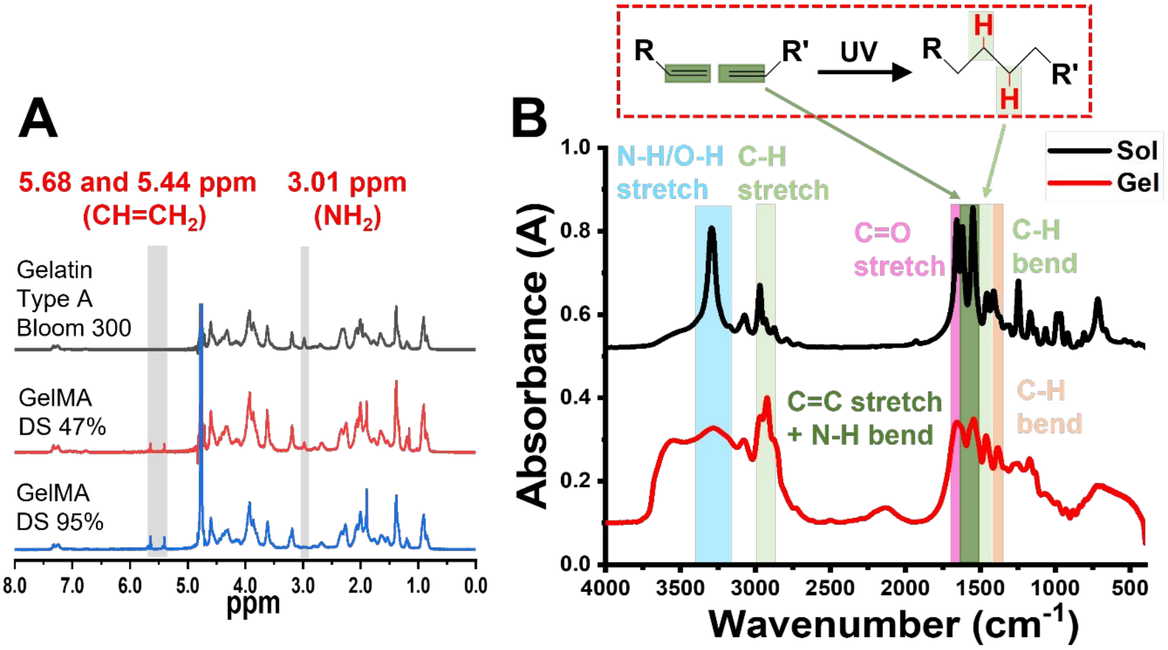


**Figure S1.** Physico-chemical characterization of PNG precursor and hydrogel. (A) GelMA with different degree of substitution (DS) was analyzed using $^1$H NMR spectroscopy. Samples were prepared in $D_2O$. (B) The FT-IR spectra of PNIPAM-GelMA (PNG′4) precursor and hydrogel confirmed successful crosslinking.

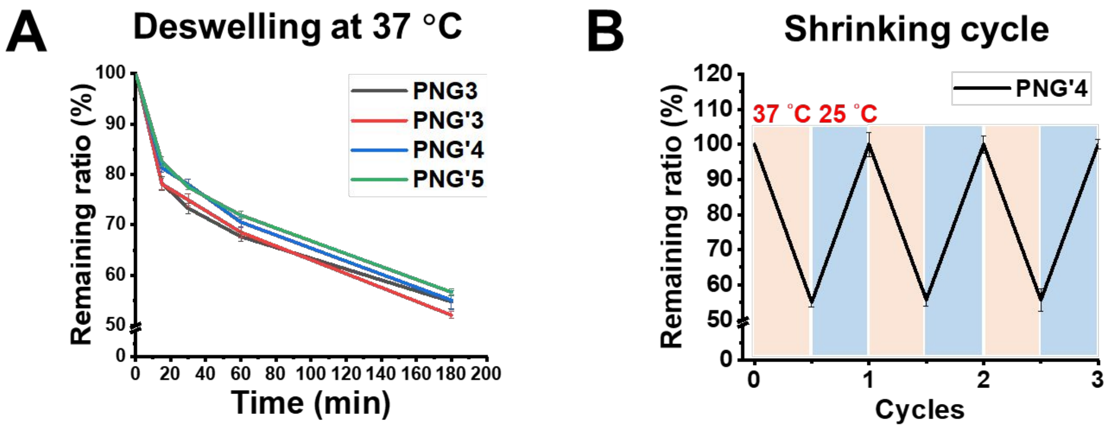


**Figure S2.** Thermoresponsive property of PNG hydrogels. (A) The remaining weight was quantified by the deswelling of various PNG hydrogels after immersion in 37 °C water. (B) Thermoresponsive deswelling and reswelling cycles of PNG′4 hydrogel were conducted for over three rounds.

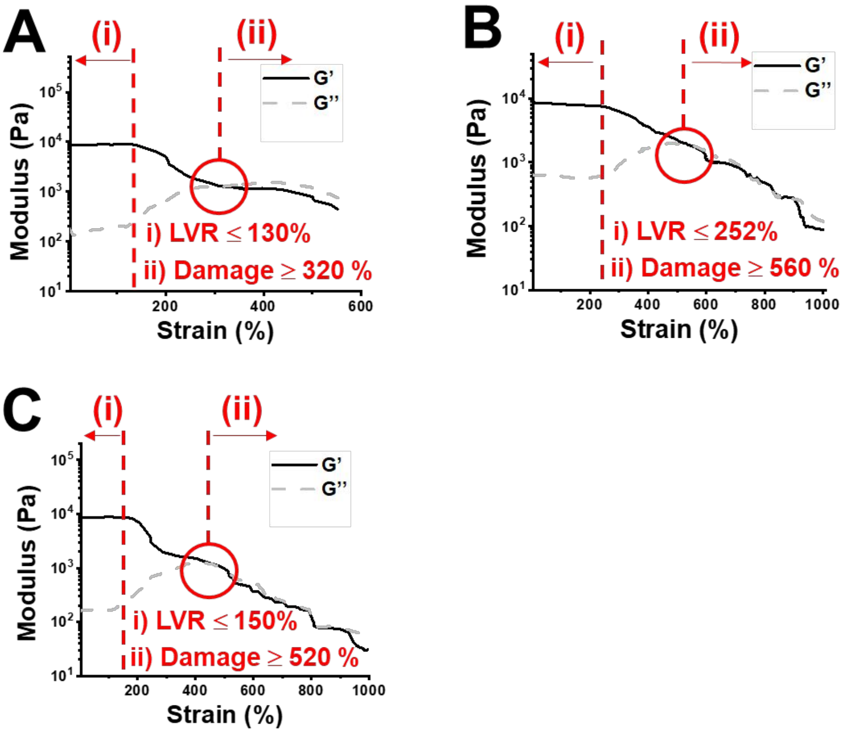


**Figure S3.** Rheological properties of post-UV PNG hydrogels. The strain sweep experiment of PNG hydrogels was conducted at 1 Hz and 25 °C, with dynamic strains ranging from 0.1% to 1000%. (A) The damage strain of PNG3 hydrogel was observed at 320%. The LVR region was observed up to 130% strain. (B) The damage strain of PNG′3 hydrogel was 560%. The LVR region was up to 252% strain. (C) The damage strain of PNG′5 hydrogel was 520%. The LVR region was up to 150% strain. Note that in (B) and (C), the hydrogel after the critical damage strain showed *G*' rather close to *G*" up to ~900% strain.

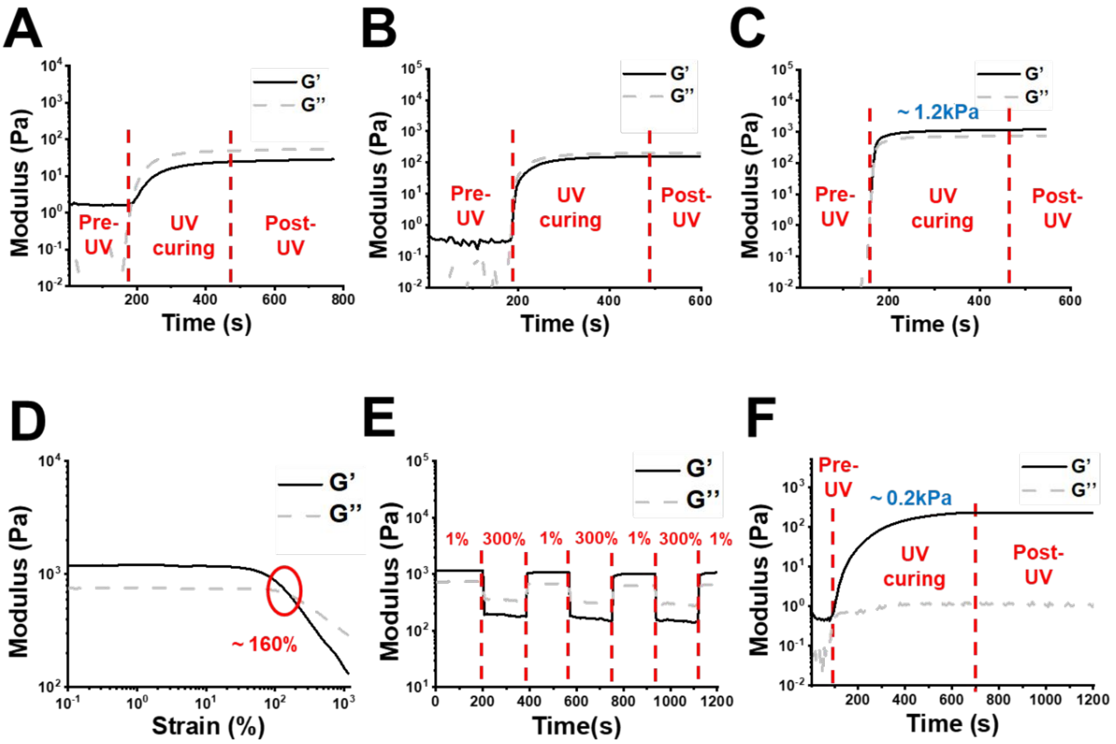


**Figure S4.** Rheological properties of pre-UV pure NIPAM solutions, post-UV pure PNIPAM hydrogels with different concentrations of PNIPAM, and post-UV 4 wt% GelMA hydrogel. (A) The time sweep experiment upon UV irradiation for 10 wt% NIPAM solution, measured at 25 °C, 1 Hz, and 1% strain. (B) The time sweep experiment upon UV irradiation for 15 wt% NIPAM solution. (C) The time sweep experiment upon UV irradiation for 20 wt% NIPAM solution. (D) The strain sweep experiment for post-UV 20 wt% PNIPAM hydrogel, was conducted at 1 Hz and 25 °C with dynamic strain ranging from 0.1% to 1000%. The damage strain was observed at 160%. (E) The damaging-healing cycle of post-UV 20 wt% PNIPAM hydrogel, measured dynamic strain alternating between 1% and 300%. The measurement was taken at 25 °C and 1 Hz. (F) The time sweep experiment for 4 wt% GelMA (DS 47%) solution upon UV irradiation.

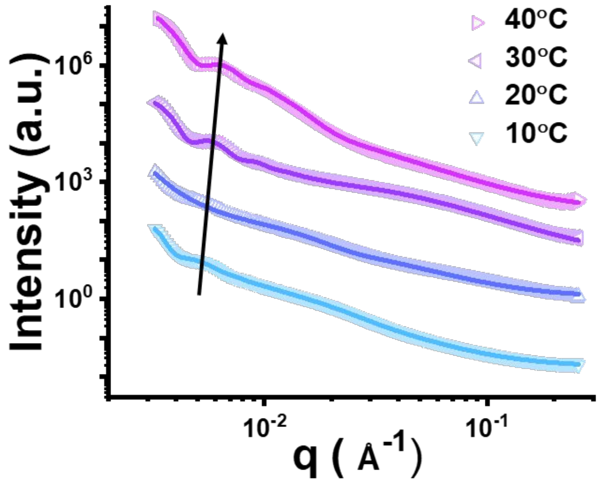


**Figure S5.** The SAXS profiles and fitting results for the PNG′4 hydrogel are shown for temperatures ranging from 10 °C to 40 °C. The scattering data (symbols) and fitting curves in low-q region (0.005–0.006 $Å^{-1}$) are vertically shifted and become more pronounced as the temperature increases, as indicated by the arrow.

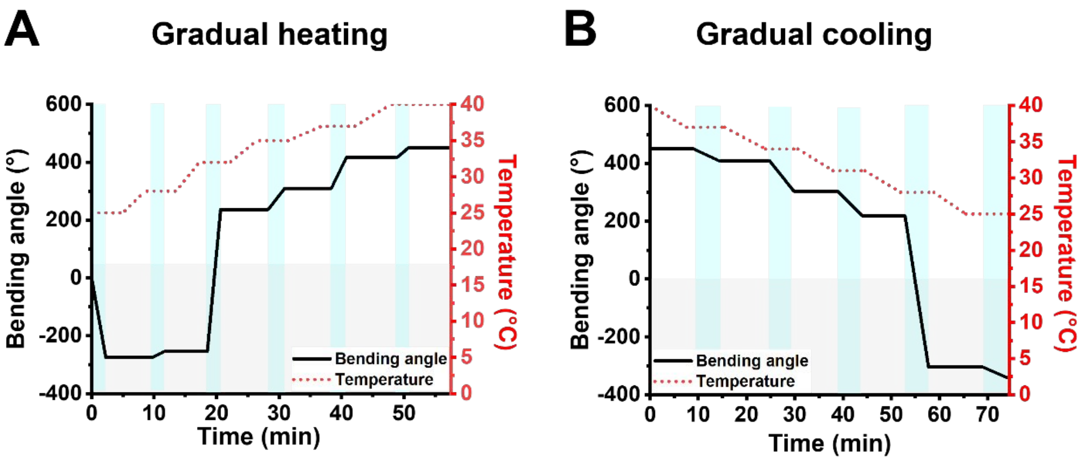


**Figure S6.** Bending response of the bilayer hydrogel actuator under stepwise temperature modulation. Light blue regions indicate response time intervals after thermal equilibration, and the gray background marks the region where bending angles are negative. (A) After immersion in 25 °C water, the actuator initially exhibits a negative bending angle. The angle gradually becomes positive as the temperature increases and reaches the transition point near 32 °C. (B) During cooling, the bending angle gradually decreases and becomes negative below approximately 32 °C.

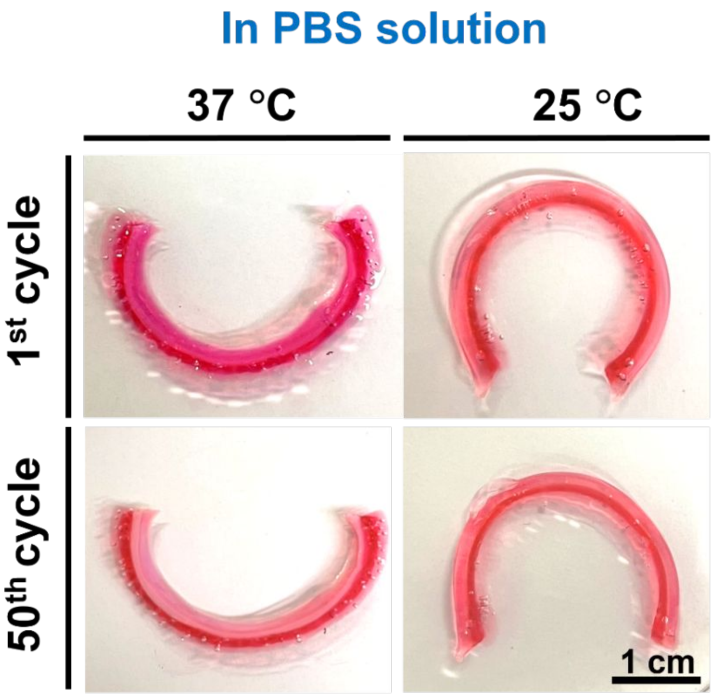


**Figure S7.** Extended cyclic actuation of a PNG′4/GelMA bilayer hydrogel actuator in PBS solution. To facilitate visualization of deformation, the passive layer was stained with Safranin-O (scale bar = 1 cm). Images show the appearance of the actuator after the first and fiftieth thermal cycles between 25 °C and 37 °C. Reversible bidirectional bending was preserved after 50 cycles, with only a modest decrease in bending amplitude relative to the first cycle.

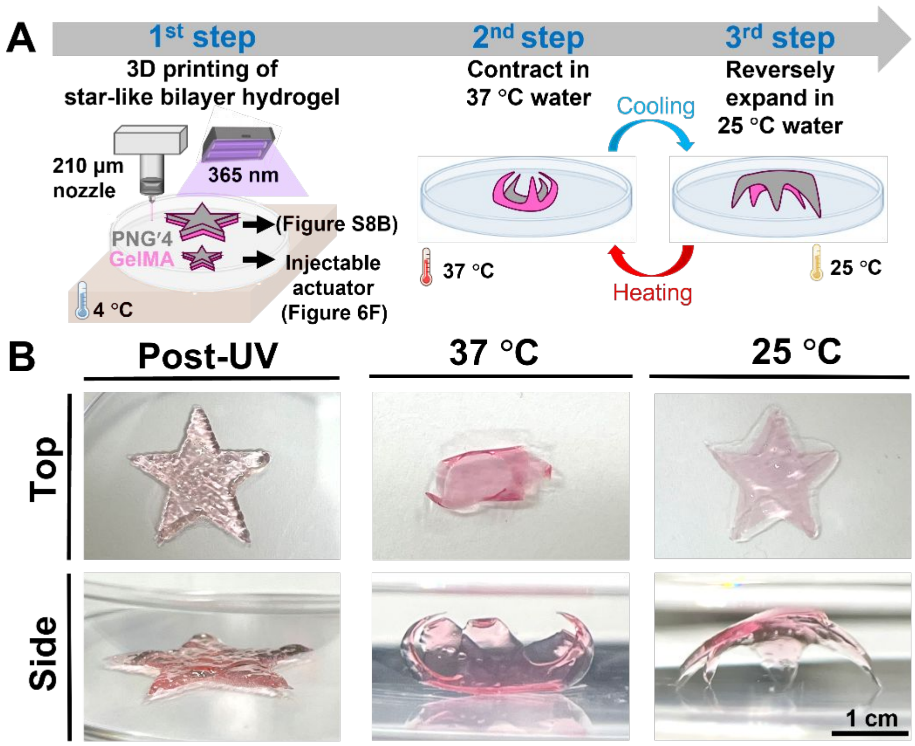


**Figure S8.** Images showing the shape deformation of the bilayer hydrogel actuators. The passive layer in both bilayer hydrogels was stained with Safranin-O for visualization (scale bar = 1 cm). (A) Schematics for the fabrication process of the larger and smaller star-like bilayer hydrogel actuators. (B) Top and side views of a star-like bilayer hydrogel actuator made by 3D printing. The star-like bilayer hydrogel (PNG′4/GelMA) contracted into a flower-like structure in 37 °C water and reversely expanded into a spider-like form in 25 °C water.

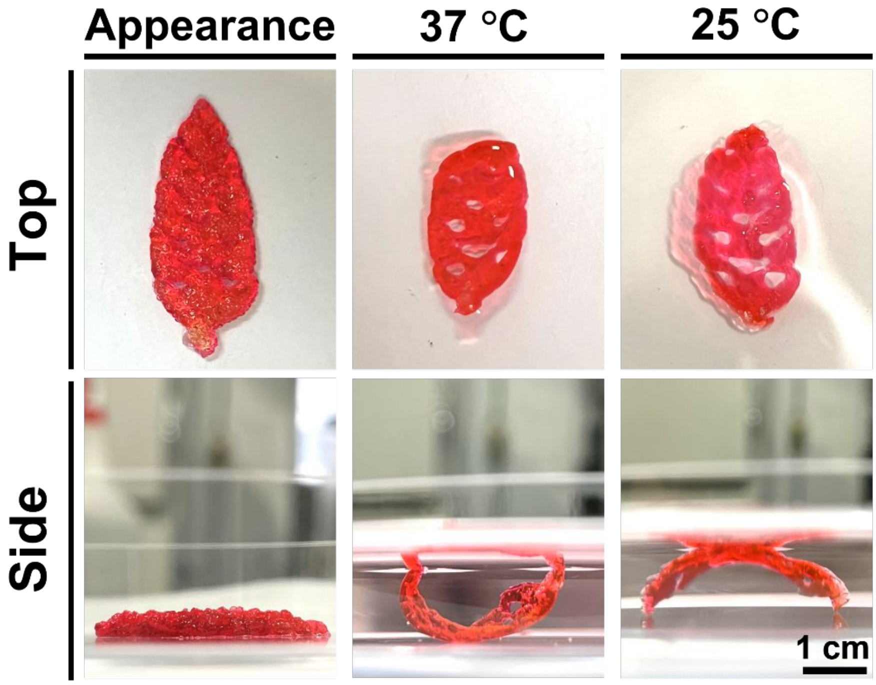


**Figure S9.** Images showing the shape deformation of a leaf-like bilayer hydrogel actuator fabricated by 3D printing. The passive layer was stained with Safranin-O for visualization (scale bar = 1 cm). The actuator preserved its fine leaf-like geometry after printing and exhibited reversible bending between 25 °C and 37 °C in water.

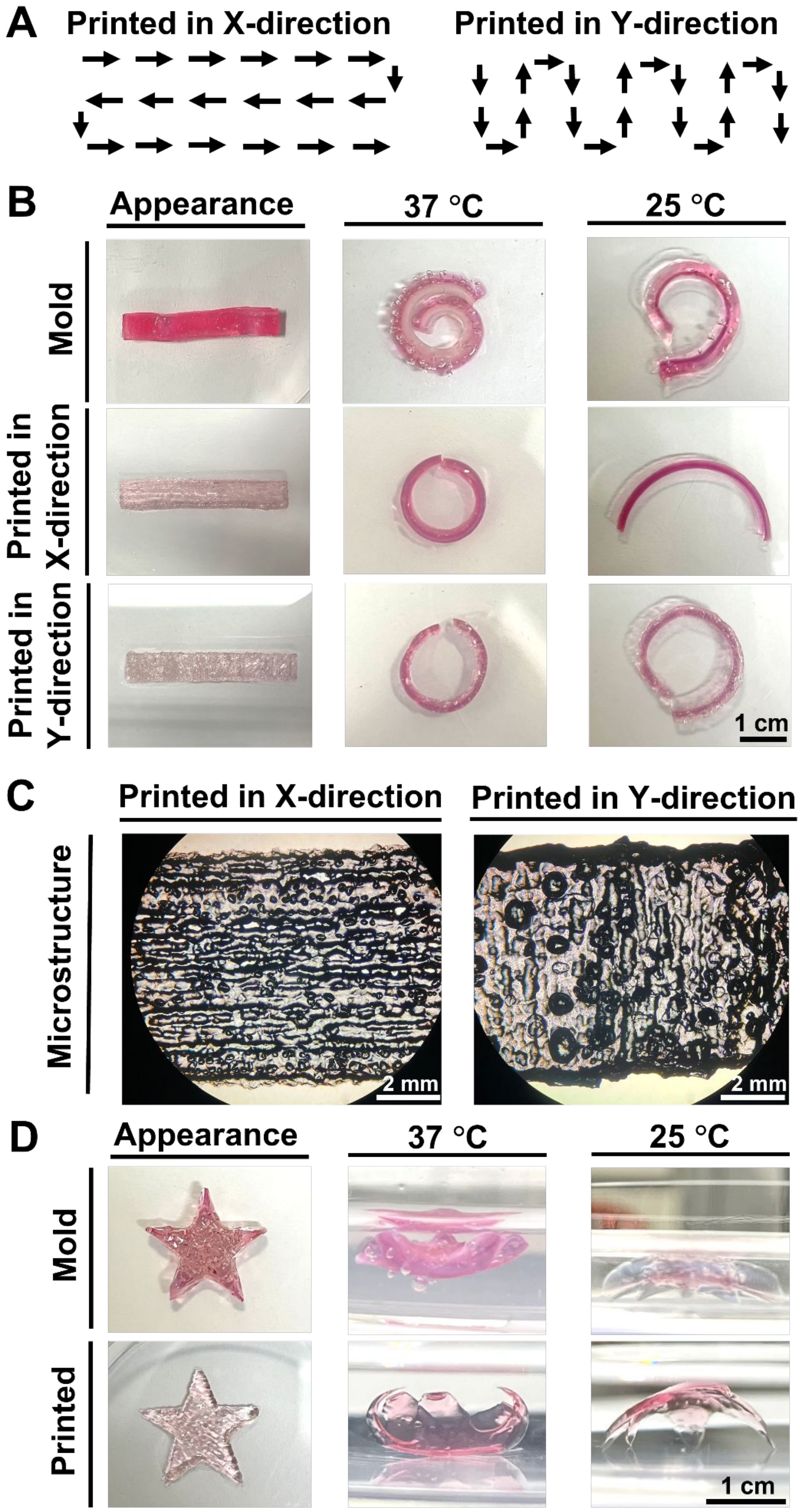
A
Printed in X-direction
Printed in Y-direction
B
Appearance
37 °C
25 °C
Mold
Printed in X-direction
Printed in Y-direction
1 cm
C
Printed in X-direction
Printed in Y-direction
Microstructure
2 mm
2 mm
D
Appearance
37 °C
25 °C
Mold
Printed
1 cm

**Figure S10.** Appearance and bending behavior of bilayer hydrogel actuators fabricated by molding and 3D printing to evaluate possible printing-induced directionality effects. The passive layer in all actuators was stained with Safranin-O for visualization. (A) Schematic illustration of the printing path for strip-shaped bilayer hydrogels, printed along either the X- axis or Y-axis. (B) Photographs showing bidirectional actuation of molded and 3D-printed strip-shaped actuators in water at 25 °C and 37 °C. The hydrogel strip printed along the X-axis showed a smaller bending angle at 25 °C. (C) Top-view optical micrographs of UV-crosslinked strips printed along the X-axis and Y-axis. Both prints displayed similar surface porosity with layers aligned parallel to the printing direction. (D) Star-like bilayer actuators fabricated by molding and 3D printing displayed comparable bidirectional bending behavior in 25 °C and 37 °C water. Scale bars are 1 cm in (B) and (D), and 2 mm in (C).

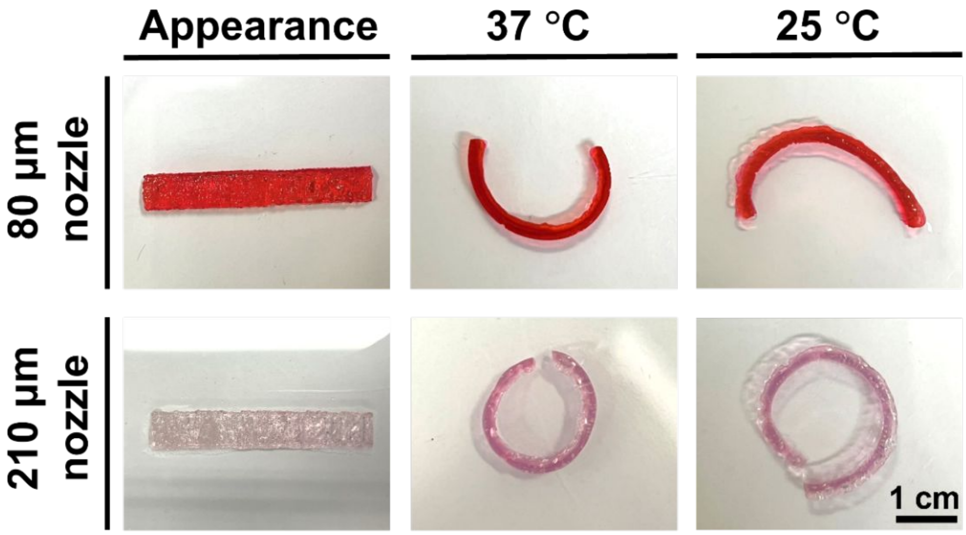


**Figure S11.** Appearance and bending behavior of strip-shaped PNG′4/GelMA bilayer actuators printed with different nozzle sizes to evaluate resolution effects. The passive layer in both strips was stained with Safranin-O for visualization (scale bar = 1 cm). The strip printed with an 80 μm nozzle showed smaller bending angles than the strip printed with a 210 μm nozzle at 25 °C and 37 °C.

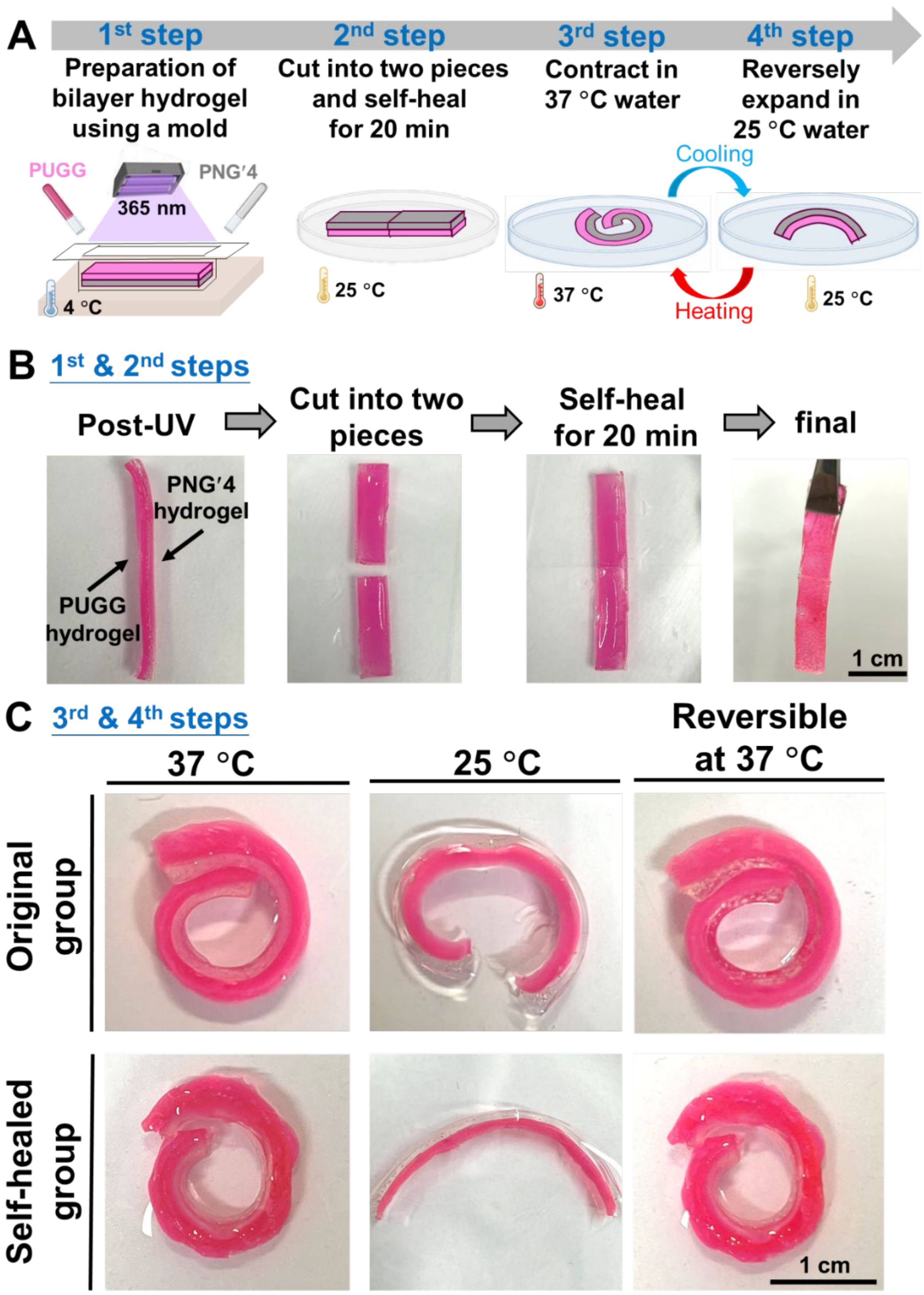


**Figure S12**. The actuation performance of the self-healable bilayer hydrogel actuators was evaluated. The actuator was prepared using PNG′4 hydrogel as the active layer and another self-healable PUGG hydrogel as the passive layer[31]. The passive layer was stained with Safranin-O for visualization (scale bar = 1 cm). (A) Schematics for the fabrication process, self-healing, and reversible actuation of the bilayer hydrogel actuator. (B) The macroscopic self-healing behavior of bilayer hydrogel actuator was observed by cutting the actuator into two halves and bringing the cut surfaces together for 20 min to facilitate self-healing. After healing, the hydrogel remained intact at the incision site when lifted with

tweezers. (C) Images illustrate the bending responses of the PNG′4/PUGG bilayer actuator in the original/intact group and the cut/self-healed group (refer to **Table S6**). During cyclic bending process in 37 °C and 25 °C water, the actuator demonstrated bidirectional bending toward the passive layer in 25 °C water both before and after self-healing. The self-healed group exhibited slightly smaller bending angle compared to the original group.

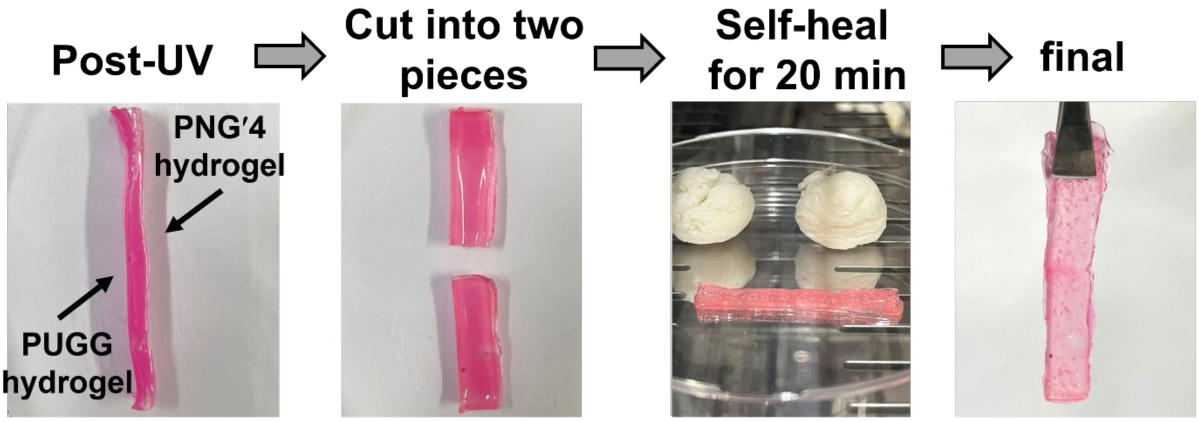


**Figure S13.** Macroscopic self-healing behavior of the PNG′4/PUGG bilayer hydrogel actuator at physiological temperature (37 °C). The actuator was first cut into two halves, then tightly rejoined and incubated in a humidified environment at 37 °C for 20 min to facilitate self-healing. After healing, the hydrogel remained intact and could be lifted without rupture at the incision site.

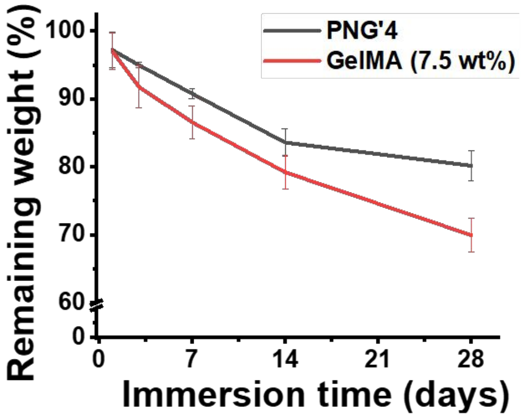


**Figure S14.** The in vitro degradation profiles of GelMA and PNG′4 hydrogels. The study was conducted in 37 °C PBS for a period of 28 days.

**Reference**

[1] Huang, Y. C.; Cheng, Q. P.; Jeng, U. S. Hsu, S. h., A biomimetic bilayer hydrogel actuator based on thermoresponsive gelatin methacryloyl-poly (N-isopropylacrylamide) hydrogel with three-dimensional printability. *ACS Applied Materials & Interfaces.* **2023**, *15*(4), 5798-5810. DOI:10.1021/acsami.2c18961
